# Similarities and Differences in the Protein Composition of Cutaneous Melanoma Cells and Their Exosomes Identified by Mass Spectrometry

**DOI:** 10.3390/cancers15041097

**Published:** 2023-02-08

**Authors:** Magdalena Surman, Urszula Jankowska, Magdalena Wilczak, Małgorzata Przybyło

**Affiliations:** 1 Department of Glycoconjugate Biochemistry, Institute of Zoology and Biomedical Research, Faculty of Biology, Jagiellonian University, 30-387 Krakow, Poland; 2 Proteomics and Mass Spectrometry Core Facility, Malopolska Centre of Biotechnology, Jagiellonian University, 30-387 Krakow, Poland; 3 Doctoral School of Exact and Natural Sciences, Jagiellonian University, 30-348 Krakow, Poland

**Keywords:** cancer, extracellular vesicles, exosomes, LC-MS/MS, melanoma, proteomics

## Abstract

**Simple Summary:**

Proteins transferred by tumor-derived exosomes can contribute to cancer progression and/or constitute novel biomarkers of a given disease. Therefore, this study used shotgun nanoLC-MS/MS to obtain complete protein profiles of four cutaneous melanoma cell lines representing different stages of the disease and exosomes released by them. As a result, 3514 and 1234 unique proteins were identified in melanoma cells and exosomes, respectively. Specific alterations to the proteomic profiles associated with disease stages have also been reported, along with a conserved portion of their proteome that may be used by various tumor cells to promote their growth and dissemination. Such a description of the complex composition of cellular and exosomal protein and their related functions provides a deeper insight into the role of exosomes in melanoma progression. The obtained results also indicate some of the exosomal proteins that should be evaluated as potential biomarkers of circulating melanoma.

**Abstract:**

Intercellular transport of proteins mediated by extracellular vesicles (EVs)—exosomes and ectosomes—is one of the factors facilitating carcinogenesis. Therefore, the research on protein cargo of melanoma-derived EVs may provide a better understanding of the mechanisms involved in melanoma progression and contribute to the development of alternative biomarkers. Proteomic data on melanoma-derived EVs are very limited. The shotgun nanoLC-MS/MS approach was applied to analyze the protein composition of primary (WM115, WM793) and metastatic (WM266-4, WM1205Lu) cutaneous melanoma cells and exosomes released by them. All cells secreted homogeneous populations of exosomes that shared a characteristic set of proteins. In total, 3514 and 1234 unique proteins were identified in melanoma cells and exosomes, respectively. Gene ontology analysis showed enrichment in several cancer-related categories, including cell proliferation, migration, negative regulation of apoptosis, and angiogenesis. The obtained results broaden our knowledge on the role of selected proteins in exosome biology, as well as their functional role in the development and progression of cutaneous melanoma. The results may also inspire future studies on the clinical potential of exosomes.

## 1. Introduction

The most aggressive form of skin cancer is malignant melanoma, which originates from transformed melanocytes, i.e., pigment cells of neuroectodermal origin. It is a multifactorial disease driven by both genetic and environmental factors. Melanoma accounts for about 4% of skin cancer cases, but it is responsible for about 75% of all related deaths. The incidence of primary cutaneous melanoma diagnosed annually worldwide has increased significantly over several decades, reaching approximately 325,000 new cases in 2020 and accounting for 1.7% of all cancers [1,2]. In addition, the mortality rate is still very high, reaching 57,000 new deaths in 2020 [2]. Despite the introduction of modern therapies for advanced stages [3,4,5], there is an urgent need to develop new effective treatment options for melanoma. Due to the fact that melanoma is characterized by a rapid growth rate and early metastases, it is also necessary to develop new, more effective diagnostic methods based on novel biomarkers.

Melanoma cells, as with any solid tumor, are surrounded by a wide variety of cells such as cancer-associated fibroblasts (CAFs), innate and adaptive immune cells, myofibroblasts, mesenchymal stem cells (MSCs), adipocytes, endothelial cells (ECs), and pericytes, as well as by an extracellular matrix (ECM), which together form the so-called tumor microenvironment (TME); all of these components are in dynamic interplay [6,7]. Cancer cells are known to act in a paracrine manner to remodel the tumor niche and regulate the interaction within the tumor microenvironment by secreting and/or inducing growth factors and cytokines, as well as releasing extracellular vesicles (EVs), to promote tumor expansion. Over the past few decades, EVs have attracted attention as an important means of communication for cancer cells due to their ability to transfer a diverse set of molecules such as proteins, lipids, RNA, DNA, and metabolites between cells [8,9,10,11,12,13]. Importantly, the molecular composition of the EV cargo reflects the pathophysiological state of the parental cell and is able to modify the biological processes of the target cell. EVs are defined as a heterogeneous population of nanoscale lipid-bilayer structures that are divided into three major groups (i.e., exosomes; ectosomes, also called microvesicles; and apoptotic bodies) based on their biogenesis, release pathways, size, and specific protein markers [14]. Bioavailability, ease of isolation, and a wide range of biological activity have made EVs the subject of numerous studies testing the possibility of their use for diagnostic, prognostic, and therapeutic purposes, as well as in the context of a better understanding of the mechanisms accompanying the progression of melanoma. The role of EVs in melanoma progression has recently been reviewed based on a large amount of data from proteomic and transcriptomic studies [15,16].

The aim of this LC-MS/MS-based study is a comprehensive proteomic comparison of primary and metastatic melanoma cells derived from the same donor and their exosomes to reveal qualitative and quantitative changes that occur with disease progression. In addition, the selective repertoires of proteins in the EV cargo, and especially the enrichment of a specific set of proteins depending on the stage of melanoma, is discussed in terms of functional implications in cancer and clinical relevance.

## 2. Materials and Methods

### 2.1. Materials

The following antibodies were used in this study: anti-CD9 mouse monoclonal primary antibodies (clone 4A2, cat. SAB1402143) and anti-CD63 mouse monoclonal primary antibodies (clone RFAC4, cat. CBL553) from Sigma-Aldrich (St. Louis, MO, USA), as well as Hsp70 (clone C92F3A-5, cat. sc-66048) and mouse monoclonal primary antibodies for Arf6 (clone 3A-1, cat. sc-7971) from Santa Cruz Biotechnology (Dallas, TX, USA). Other reagents were obtained as listed in [17].

### 2.2. Cell Lines and Culture Conditions

WM115/WM266-4 [18], WM793 [19]/WM1205Lu [20]—isogenic pairs (primary/metastatic) of CM cell lines obtained from the ESTDAB Melanoma Cell Bank (Tübingen, Germany)—were used for LC-MS/MS analysis and exosome isolation. Cells were cultured in standard conditions (5% CO_2_, 37 °C) in GlutaMAX-I RPMI 1640 medium with 10% FBS, penicillin (100 unit/mL), and streptomycin (100 μg/mL). After reaching 80% confluence, cell cultures were passaged or conditioned media was used for exosome isolation.

### 2.3. Isolation of Exosomes and Assessment of the Purity of the Exosome Samples

Before exosome isolation, CM cells were kept for 24 h in FBS-free media. Conditioned media were then collected (approx. 200 mL per exosome sample) and centrifuged. Through centrifugations at 400× *g* (5 min, 4 °C) and 4000× *g* (20 min, 4 °C), cells and cellular debris were removed; supernatants were then concentrated by a low-vacuum filtration (LVF) procedure described by Drożdż et al. [21]. Concentrated media (approx. 2 mL) were centrifuged three times: at 7000× *g* (20 min, 4 °C), 18,000× *g* (20 min, 4 °C), and 80,000× *g* (20 min, 4 °C), to remove any larger vesicles. The final centrifugation step was performed at 150,000× *g* (90 min, 4 °C) to obtain exosome pellets. Finally, exosomes were resuspended in ice-cold PBS or in a LC-MS/MS lysis buffer.

The purity of exosome samples was analyzed by nanoparticle tracking analysis (NTA) on NanoSight LM 10 (Malvern Panalytical, Malvern, UK). Five independent records were collected for each sample (10 μL diluted to 2 mL with PBS). The mean results ± SD are presented on graphs.

In addition, immunodetection of EV markers was performed. SDS-PAGE electrophoresis and WB (western blot) for each CM cell line and exosome sample were performed as described in [22]. The chosen markers were detected using anti-CD63 (1:2000), anti-CD9 (1:2000), anti-Hsp70 (1:2000), and anti-Arf6 (1:500) primary antibodies, and anti-mouse IgG-HRP (1:400) as a secondary antibody. Chemiluminescence-based detection was done using HRP substrates and ChemiDoc Imaging System (Bio-Rad, Hercules, CA, USA).

### 2.4. LC–MS/MS Proteomics

Exosome lysis, sample preparation for mass spectrometric analysis, and LC-MS/MS analysis were performed as described in [17,23] with minor modifications. Namely, the acetonitrile gradient on the analytical column was 240 min, and the flow rate was 250 nL/min. In addition, the Q-Exactive was operated using the top 12 method. Full-scan MS spectra were acquired with automatic gain control (AGC target) of 1 × 10^6^, and the MS/MS spectra were acquired with an AGC target of 5 × 10^5^. The maximum ion accumulation times for the full MS and the MS/MS scans were 120 ms and 60 ms, respectively.

### 2.5. Analysis of Proteomic Data

Raw mass spectra were processed using MaxQuant 2.0.3.1 [24]. Peak lists were searched against the forward and reverse Swissprot_database restricted to *Homo sapiens* taxonomy (20,376 sequences; downloaded on 5 May 2022) with the use of the integrated Andromeda search engine. Fully tryptic peptides with a maximum of two missed cleavages and with at least seven amino acids were treated as valid. Cysteine carbamidomethylation was set as a fixed modification, whereas variable modifications included methionine oxidation and protein N-terminal acetylation. The precursor mass tolerance in the first search used for mass recalibration was set to 20 ppm. The main search was performed with precursor and fragment mass tolerances of 4.5 ppm and 20 ppm, respectively. The maximum false discovery rate for both peptide and protein identification were set to 0.01. The MaxLFQ label-free algorithm using a minimum ratio count of 2 was used for relative quantification and normalization. Both razor and unique peptides were used for protein quantitation. Separate batches were used to analyze samples from cells and exosomes.

The raw data were deposited via the MassIVE repository to the ProteomeXchange Consortium with the dataset identifier PXD038861.

Further analysis was performed on the Perseus platform (version 2.0.7.0) [24]. All contaminants, the proteins from the decoy database, and proteins identified only by modified peptides were excluded from the study. Quantitative analysis was performed on label-free quantification (LFQ) intensities transformed to the logarithmic scale. The student’s *t*-test was then performed with the permutation-based FDR at 0.01 and 0.05 to reveal changes in the protein abundances between different melanoma cell lines and exosomes. Only proteins with at least three valid LFQ intensity values in both compared groups were considered for statistical analysis. Proteins identified by at least two peptides, score > 10, and with a fold change of at least 1.5 (for comparison between cells) or 1.2 (for comparison between exosomes) were considered as differential. Entire protein lists are provided in Supplementary Data S2. Complete quantitative analysis is provided in Supplementary Data S3.

### 2.6. Bioinformatic Analysis

The final protein lists contained proteins identified by at least two peptides in three or four out of four biological repetitions of cellular or exosome samples. Venn diagrams, including Vesiclepedia protein overlap, and gene ontology (GO) analysis were performed with FunRich 2.0 software using the UniProt (release 2022_11) database as a reference. For each GO term, six categories with the highest protein enrichment were presented as graphs. Additionally, enrichment within selected cancer-related categories (calculated as −log10(*p*-value)) was compared between CM cells and exosome samples from primary and metastatic cells. Entire protein lists and GO data are included in Supplementary Materials Data S1. Interaction diagrams from Appendix A and Supplementary Data S3 were prepared with the use of https://string-db.org/ (accessed on 2 December 2022) Version: 11.0.

## 3. Results

### 3.1. Characterization of CM Exosome Samples

**Figure 1:**
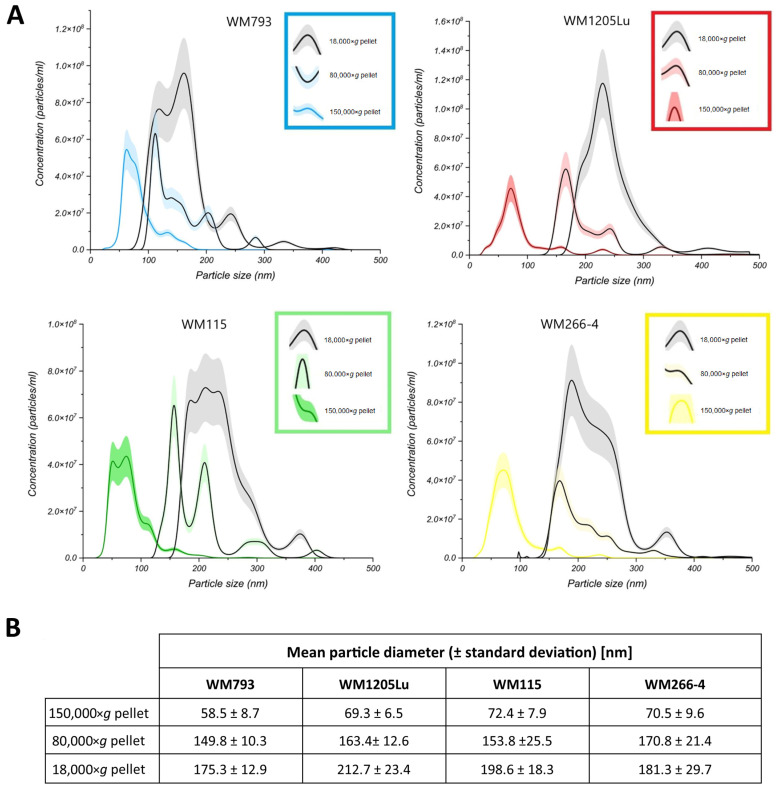
Nanoparticle tracking analysis (NTA) of pellets obtained during exosome isolation, i.e., intermediate centrifugation steps (18,000× *g* and 80,000× *g*), and of final 150,000× *g* pellet (exosomes). (**A**) The graphs show means from five independent measurements with ± standard deviation (shaded area). (**B**) Mean particle diameter ± standard deviation measured for all pellets were also presented in table. (WM115/WM266-4,WM793/WM1205Lu—isogenic pairs (primary/metastatic) of CM cell lines that were used for exosome isolation.

In the present study, a complex exosome isolation protocol based on sequential centrifugation and low vacuum filtration (LVF) was applied [21]. Nanoparticle tracking analysis (NTA) showed that the final 150,000× *g* exosome pellets contained mostly <100-nm-diameter vesicles (typical range of exosomes), with less than 5% being >100-nm-diameter vesicles (Figure 1).

**Figure 2:**
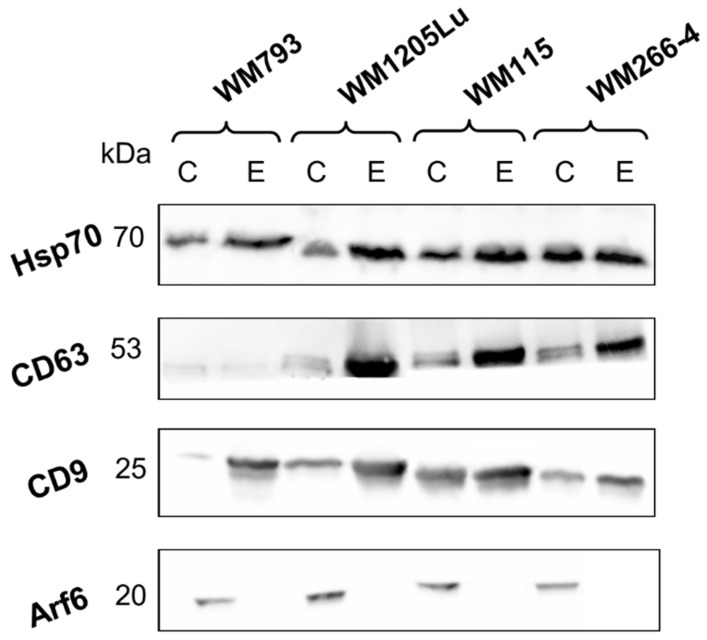
Immunodetection of extracellular vesicle (EV) markers in whole-cell protein extracts (lines C) and exosome samples (lines E). Prior to immunodetection, 50 µg of protein per line was separated by 10% SDS-PAGE and transferred into the PVDF membrane. Immunodetection was performed with the use of the following primary antibodies: anti CD9, anti-CD63, anti-Hsp70, and anti-Arf6, and anti-mouse IgG-HRP as a secondary antibody. Original blots were provided in Supplementary Data S1. WM115/WM266-4,WM793/WM1205Lu—isogenic pairs (primary/metastatic) of CM cell lines that were used for exosome isolation.

Additionally, enrichment of exosomal protein markers (CD9, CD63, and Hsp70) was confirmed in each exosome sample compared to whole-cell protein extracts (Figure 2). On the other hand, exosome samples were depleted of Arf6, the protein involved directly in ectosome biogenesis but not in exosome biogenesis. The evidence presented allows us to consider the isolated EV samples to be highly enriched in exosomes and the ectosome contamination to be negligible.

### 3.2. Identified Proteins of CM Cells and Exosomes and Their Functional Classification

**Figure 3:**
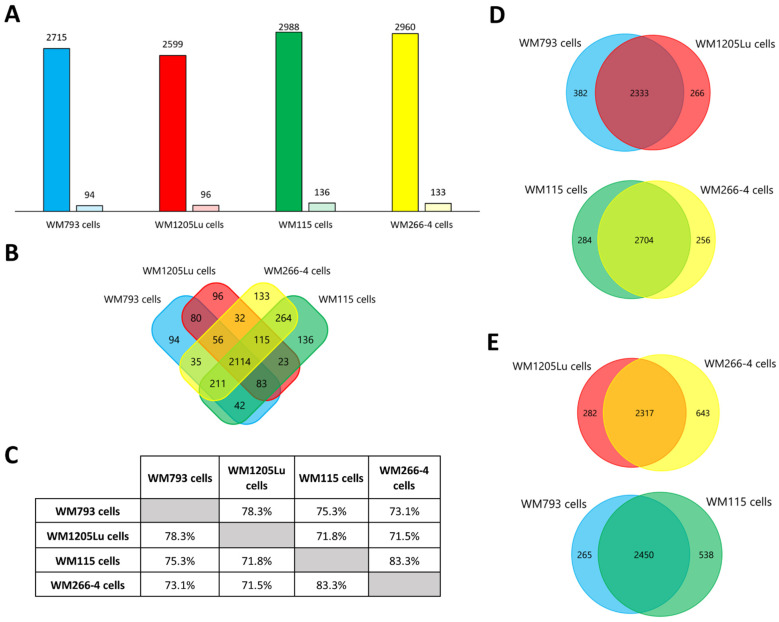
Qualitative analysis of proteins identified in CM cells by LC-MS/MS. (**A**) Number of proteins identified (by at least two peptides) in at least three out of four biological replicates of each CM cell sample (left bar) and the number of unique proteins (right bar). (**B**) Number of proteins shared between given CM cell lines. (**C**) Percentage of proteins shared between given CM cell lines. Number of proteins shared between isogenic (**D**) and primary or metastatic CM cell lines (**E**). WM115/WM266-4,WM793/WM1205Lu—isogenic pairs (primary/metastatic) of CM cell lines that were analyzed. Complete protein lists are presented in Supplementary Data S2.

**Figure A1:**
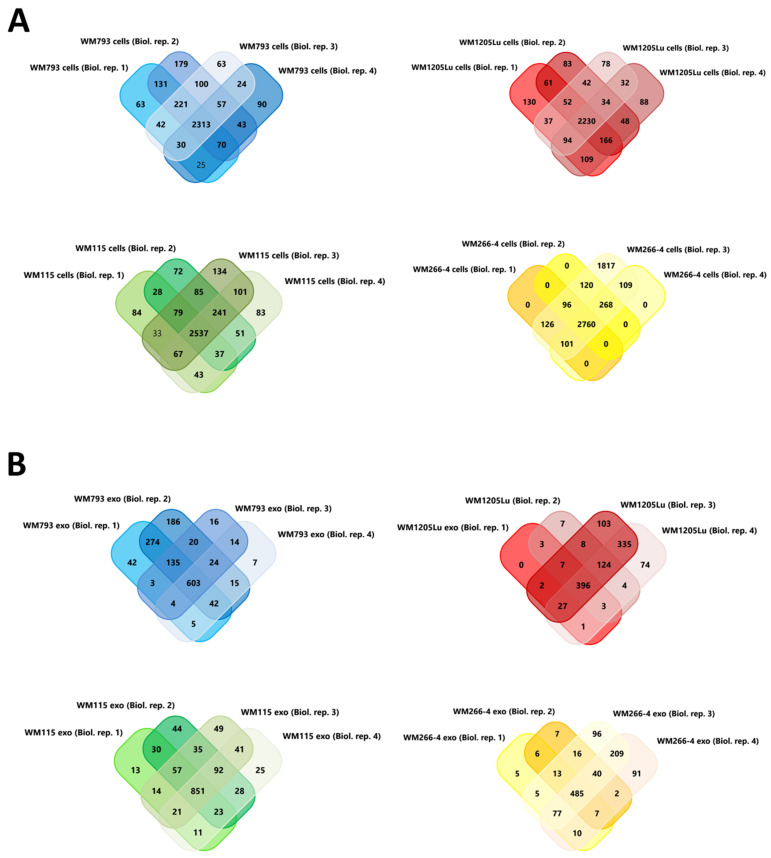
Venn diagrams illustrating the number of proteins shared between biological replicates of given CM cell (**A**) and ectosome (**B**) samples.

Protein profiles for four CM cell lines and exosomes released by these cells were obtained using the gel-free nanoLC–MS/MS shotgun proteomic approach. Four biological replicates of each cellular/exosomal sample were analyzed, and only proteins identified in at least three replicates (and with at least two peptides) were considered for further analyses. Variability of protein detection across replicates was presented (Figure A1). A total of 3514 proteins were identified for all CM cell lines analyzed (Figure 3A, complete protein lists in Supplementary Data S2). In terms of individual cell lines, a greater number of proteins were identified for primary WM793 and WM115 cells compared to their isogenic metastatic equivalents, i.e., WM1205Lu and WM266-4 cells, respectively. In addition, 2114 proteins (approx. 60% of all identified proteins) were identified for all CM cell lines analyzed (Figure 3B). More than 70% similarity in protein composition was observed for all possible pairings of cell lines analyzed (Figure 3C–E). The greatest similarity was observed between isogenic pairs, i.e., WM115 and WM266-4 (83.3% of shared proteins), and WM793 and WM1205Lu (78.3% of shared proteins) cells, which can be attributed to their common genetic background.

**Figure 4:**
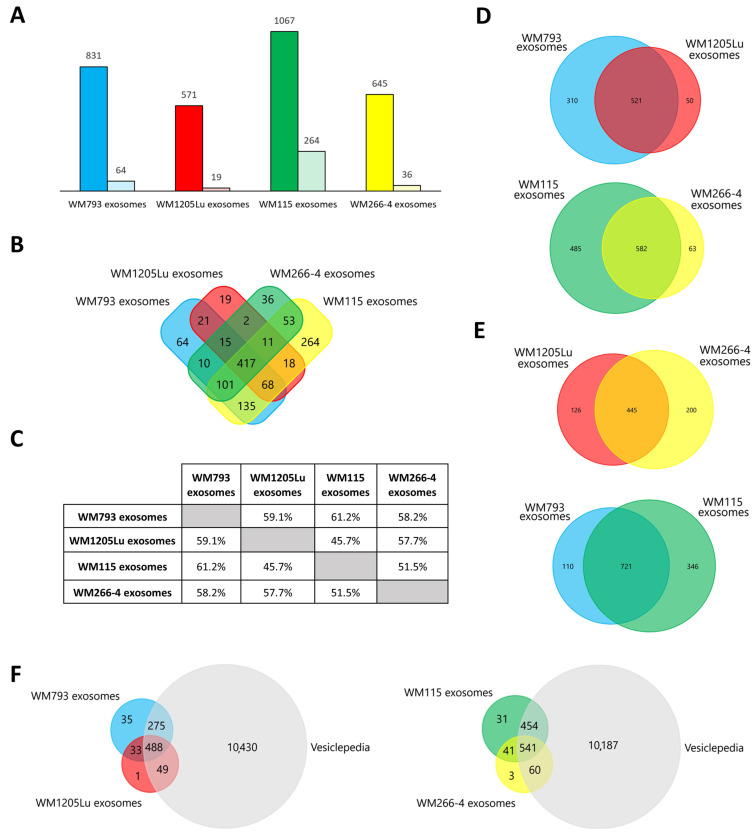
Qualitative analysis of proteins identified in CM-derived exosomes by LC-MS/MS. (**A**) Number of proteins identified (by at least two peptides) in at least three out of four biological replicates of each CM exosome sample (left bar) and the number of unique proteins (right bar). (**B**) Number of proteins shared between exosomes derived from given CM cell lines. (**C**) Percentage of proteins shared between given CM-derived exosome samples. Number of proteins shared between exosomes derived from isogenic (**D**) and primary or metastatic CM cell lines (**E**). (**F**) Venn diagram illustrating protein overlap between isolated ectosomes and Vesiclepedia database as a reference. WM115/WM266-4,WM793/WM1205Lu—isogenic pairs (primary/metastatic) of CM cell lines that were used for exosome isolation. Complete protein lists are presented in Supplementary Data S2.

A total of 1234 unique proteins were identified in CM-derived exosomes (Figure 4A, complete lists of proteins are given in Supplementary Data S2). Exosomes released by primary WM793 and WM115 cells were characterized by a greater number of identified proteins than their isogenic metastatic pairs—WM1205Lu and WM266-4, respectively. It is possible that metastatic cell lines retain more proteins (rather than secrete them) due to their overactive metabolism. Exosomes derived from primary WM115 cells had the highest number of proteins identified, 1067, while exosomes derived from metastatic WM1205Lu cells had the lowest number of proteins, 571. The number of unique proteins for a given exosome sample was between 19 (WM1205Lu-derived exosomes) and 264 (WM115 exosomes). In contrast, 417 proteins were identified in exosomes from all CM cell lines (Figure 4B), accounting for 33.8% of all identified exosomal proteins compared to approximately 60% for cell-derived samples. The highest similarity of the proteome was observed between exosomes derived from two primary cell lines, i.e., WM793 and WM115 (61.2%), and the lowest for WM1205Lu and WM115 exosomes (45.7%) (Figure 4C–E). In addition, the similarity was high for exosomes of metastatic origin (WM1205Lu and WM266-4), i.e., 57.7%. This suggests that the protein composition of exosomes may be more dependent on the disease stage than on the genetic background of the parental cell (which was observed for CM cell samples). Additionally, all proteins identified in exosome samples were searched against the Vesiclepedia database (Figure 4F), which collects proteomic data from multiple EV-oriented studies. There was a significant overlap between the proteins identified in the present study and the Vesiclepedia dataset. which supports their vesicular origin instead of being part of cell-derived contamination.

**Figure 5:**
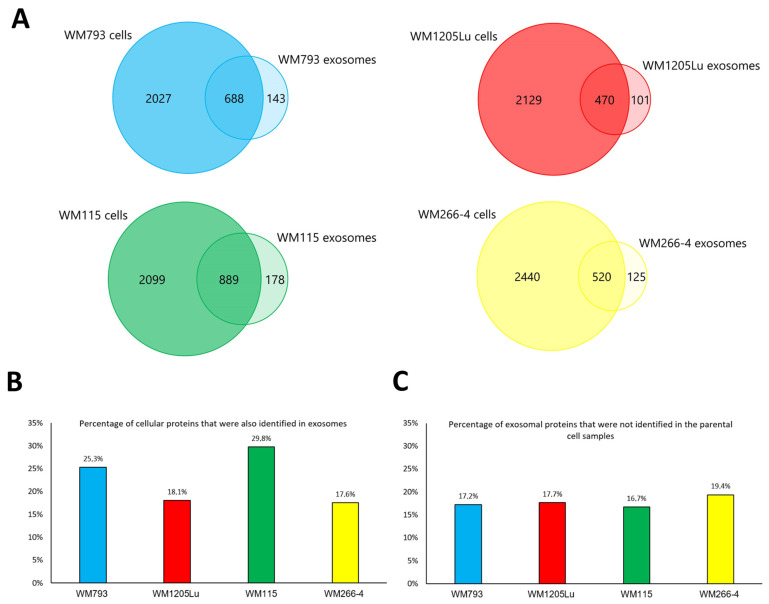
Qualitative analysis of proteins identified in CM cell lines and CM-derived exosomes by LC-MS/MS. (**A**) Number of proteins common for exosome samples and the cell line of their origin. Only proteins identified in at least three out of four biological replicates (by at least two peptides) were considered for analyses. (**B**) Percentage of proteins that were identified in CM cell lines and then in the exosome sample derived thereof. (**C**) Percentage of proteins that were identified in CM exosomes but not in the parental cell samples. Exosomes were isolated from WM115/WM266-4 and WM793/WM1205Lu cells—isogenic pairs (primary/metastatic) of CM cell lines.

Furthermore, the proteomes of CM cells and exosomes were compared (Figure 5). For each cell line, from ca. 17% to almost 30% of their proteins were also identified in exosomes. This suggests that the sorting of protein into exosomes is a highly regulated process involving only a very specific set of proteins. On the other hand, between 16% to 20% of proteins identified in exosome samples were not identified in cells. Since exosomal proteins must be derived from the cell, the lack of identification in CM cells may be due to their low abundance in total cellular proteomes and sufficient enrichment in exosomes.

**Figure 6:**
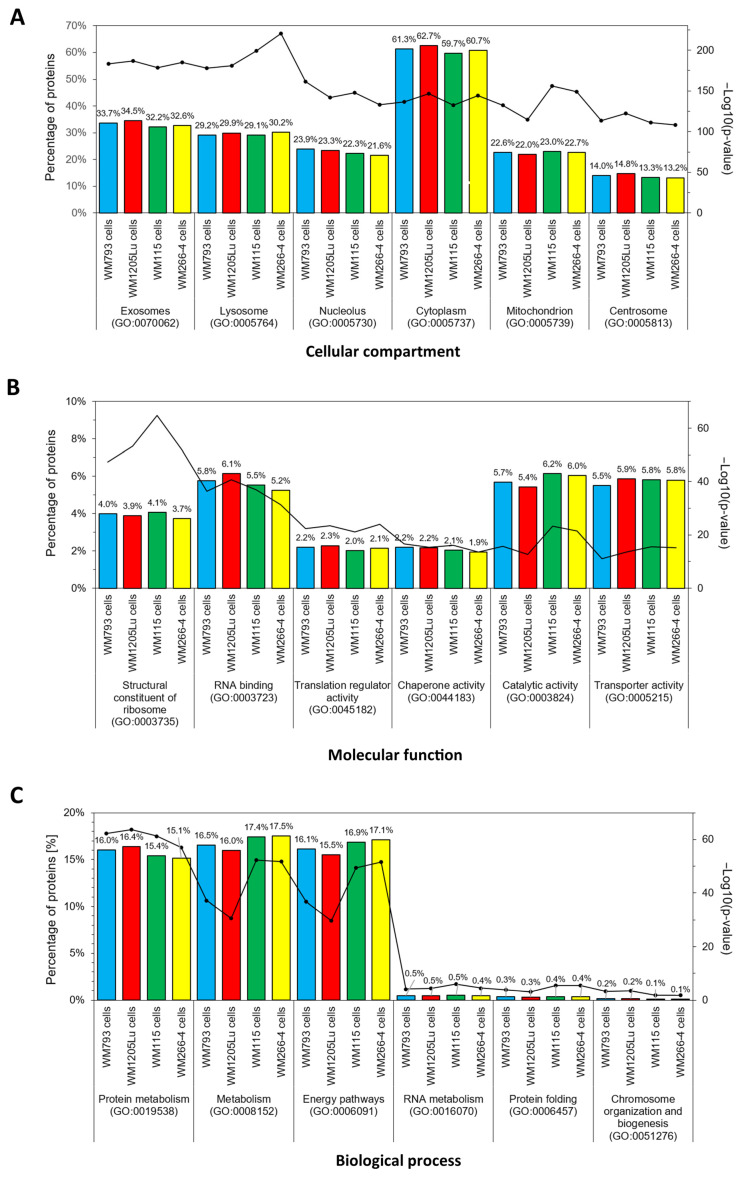
GO analysis regarding “Cellular compartment” (**A**), “Molecular function” (**B**), and “Biological process” (**C**) of proteins identified in CM cell lines. FunRich 3.1.3. software with the UniProt (release 2022_11) database was used, and six categories with the highest statistical significance of protein enrichment (*p* < 0.001) were presented in graphs. Complete results are presented in Supplementary Data S2. WM115/WM266-4,WM793/WM1205Lu—isogenic pairs (primary/metastatic) of CM cell lines that were analyzed.

Gene ontology (GO) analysis was then performed to group proteins identified in CM cells and exosomes by the cellular compartment, molecular function, and biological processes. The six categories with the highest statistical significance of protein enrichment within the category (*p* < 0.001) were selected for each analysis. Complete protein lists are shown in Supplementary Data S2. Similar GO patterns were observed for each CM cell line (Figure 6). The most numerous groups of proteins were associated with the cytoplasm (up to 62.7% of identified proteins). Moreover, up to 34.5% of proteins identified in CM cells were connected to exosomal origin. GO analysis of molecular function showed that the identified proteins were mainly associated with catalytic (up to 6.2%) or transporter activity (up to 5.9%), and RNA binding (up to 6.1%). A significant abundance of structural proteins were also identified. Furthermore, the analysis of the biological function of cellular proteins pointed out that identified proteins are mostly involved in metabolism, especially protein metabolism, and in various energy pathways.

**Figure 7:**
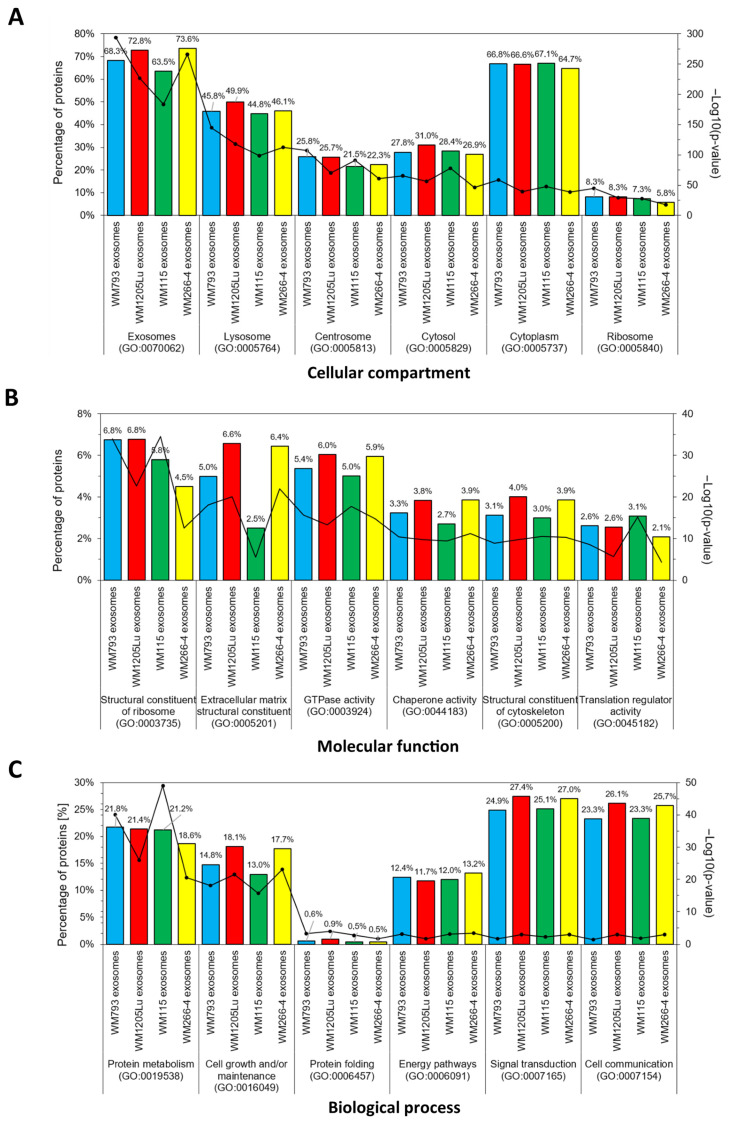
GO analysis regarding “Cellular compartment” (**A**), “Molecular function” (**B**), and “Biological process” (**C**) of proteins identified in CM-derived exosomes. FunRich 3.1.3. software with the UniProt (release 2022_11) database was used, and six categories with the highest statistical significance of protein enrichment (*p* < 0.001) were presented in graphs. Complete results are presented in Supplementary Data S2. WM115/WM266-4,WM793/WM1205Lu—isogenic pairs (primary/metastatic) of CM cell lines that were used for exosome isolation.

GO analysis of CM-derived exosomal proteins showed that up to 73.6% of the identified proteins had exosomal origin and up to 67.1% were associated with the cytoplasm (Figure 7). This observation is consistent with exosome biogenesis, which involves blebbing of endosomal membranes and creating multivesicular bodies, which later fuse with the cell membrane and give rise to exosomes. In addition, GO analysis for the molecular function revealed the greatest enrichment in categories such as GTPase, chaperone, and translation regulatory activity. CM exosomal proteins function as structural constituents of ribosomal and extracellular matrices. In terms of biological processes, the proteins identified were involved in cell communication (over 23%), signal transduction (over 24%), protein metabolism (over 18%), and cell growth and/or maintenance (over 13%). Metastatic WM266-4- and WM1205Lu-derived exosomes showed higher enrichment in several categories than their primary counterparts, including cell growth and/or maintenance, cell communication, signal transduction, GTPase activity, etc. This may reflect higher prometastatic potential compared to exosomes from primary cells.

### 3.3. Functional Similarities and Differences in the Protein Composition of CM Cells and CM-Derived Exosomes

**Figure 8:**
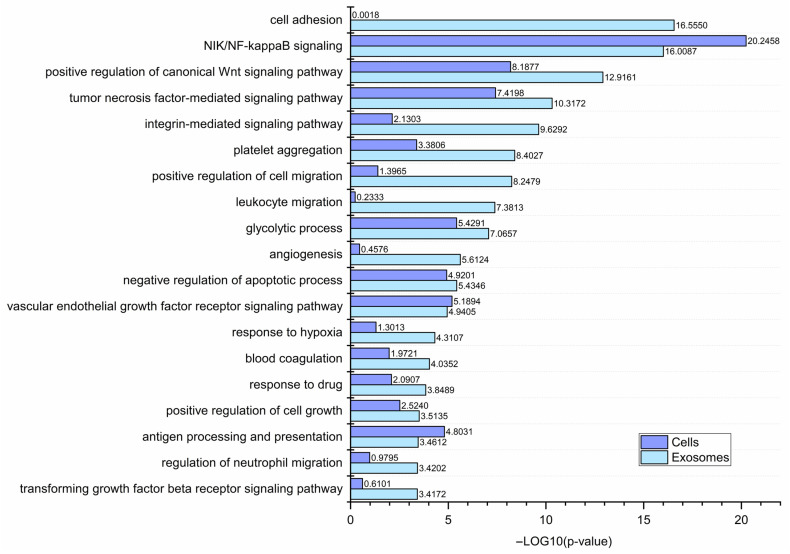
Gene ontology annotations by the biological process of all proteins identified in CM cells (3514 proteins) or CM-derived exosomes (1234 proteins) performed via the UniProt (release 2022_11) database. Enrichment significance scores (i.e., −log10(*p*-value)) within the chosen cancer-related categories for CM cells or CM-derived exosomes are presented in the graph. WM115/WM266-4,WM793/WM1205Lu—isogenic pairs (primary/metastatic) of CM cell lines that were used for LC-MS/MS analysis and exosome isolation. Complete results are presented in Supplementary Data S2.

An increasing number of studies are focusing on the role of EVs in various diseases, including cancer. Tumor-derived EVs facilitate the transfer of biologically active molecules that can regulate the function of the recipient cell at various levels. To gain insight into how CM-derived exosomes might affect recipient cells, GO analysis was performed using the lists of all proteins identified in CM cells (3514 proteins) and CM-derived exosomes (1234 proteins). Selected cancer-related GO categories and their enrichment significance score (i.e., −log10(*p*-value)) were presented in Figure 8.

Considering categories with *p* < 0.001, the analyzed CM cells were enriched in proteins related to antigen processing and presentation, negative regulation of apoptosis, platelet aggregation, and glycolysis. All these processes are enhanced in cancer cells, which tend to avoid immune surveillance, escape apoptosis, and contribute to the prothrombotic state, as well as rely on aerobic glycolysis for ATP generation. Numerous other categories for CM cells included several signaling pathways, i.e., the NIK/NF-kappaB pathway, the Wnt pathway, the tumor necrosis factor-mediated pathway, and the vascular endothelial growth factor receptor (VEGFR) signaling pathway. These are all known to be altered in cancer and involved in key steps in the metastatic cascade, such as angiogenesis.

Since exosomes (and other EVs) largely reflect the composition of parental cells, CM-derived exosomes isolated in the present study also showed enrichment in selected cancer-related GO categories. Notably, for most categories, enrichment was more significant for CM-derived exosomes than for CM cells. This includes categories that were not enriched (*p* > 0.001) for CM cells, such as cell growth, cell adhesion, cell migration, response to hypoxia, blood coagulation, or angiogenesis. In addition, CM-derived exosomes showed enrichment in categories related to immune cell migration, suggesting their involvement in the modulation of the immune response against melanoma tumors. Finally, the GO category “drug response” was enriched for CM-derived exosomes, indicating their possible role in the phenomenon of multidrug resistance.

To characterize the changes in the cellular and exosomal proteome that occur during disease progression, the proteins identified in CM cells and CM-derived exosomes were qualitatively and quantitively compared within isogenic pairs (same donor) of cell lines (that is, WM793 vs. WM1205Lu and WM115 vs. WM266-4, respectively). Table 1 lists the proteins that were identified by all four replicates in WM793 (79 proteins) but not in 1205 Lu cells and vice versa (38 proteins). Smaller discrepancies in the number of unique proteins were observed for WM115 and WM266-4 cells, i.e., 31 and 40 proteins, respectively (Table 2). Considering exosomes, 58 proteins were identified in WM793-derived exosomes but not in WM1205Lu-derived exosomes, with only two proteins unique to WM1205Lu-derived exosomes (Table 3). Similarly for the second isogenic pair, exosomes derived from WM115 cells contained more unique proteins (79 proteins) than exosomes derived from WM266-4 (34 proteins) (Table 4). This suggests that as the disease progresses, tumor cells tend to sort fewer proteins into exosomes, possibly to preserve/increase their own metastatic potential. Another possibility is that metastatic cells sort fewer proteins into exosomes but increase the abundance of proteins crucial for cancer progression.

**Figure A2:**
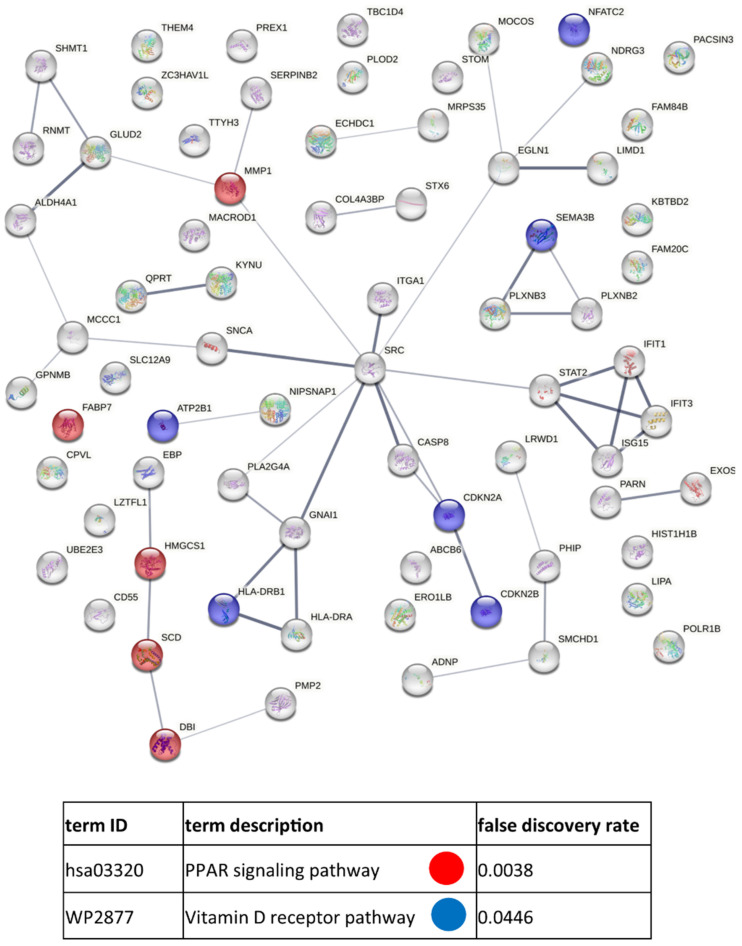
Diagram of functional protein association networks prepared with the use of STRING v. 11.0 software for proteins identified in WM793 cells but not found in any replicates of WM1205Lu cells. The selected, strongly represented pathways are presented as an interactome. A complete analysis is presented in Supplementary Data S3.

**Figure A3:**
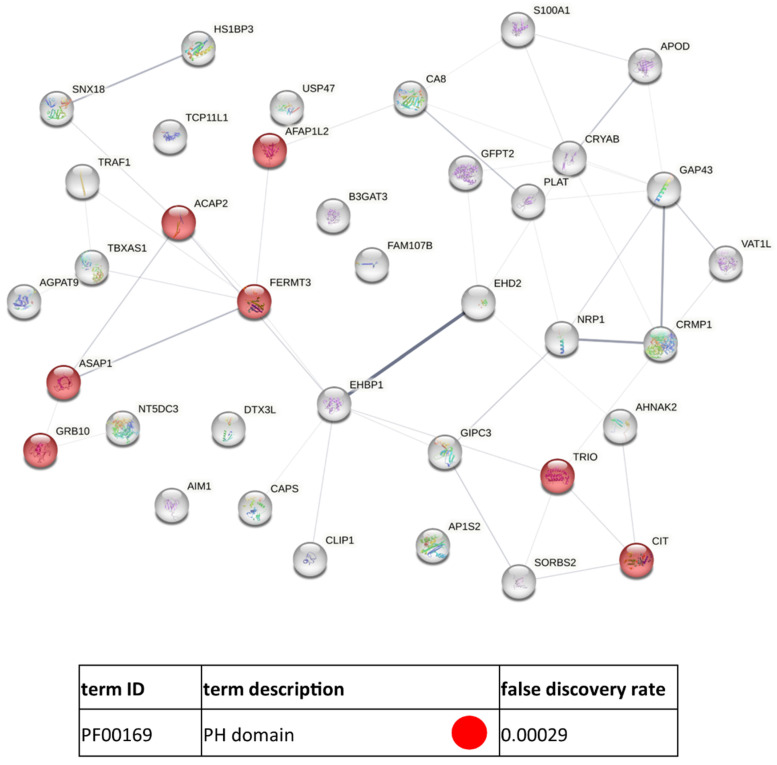
Diagram of functional protein association networks prepared with the use of STRING v. 11.0 software for proteins identified in WM1205Lu cells but not found in any replicates of WM793 cells. The selected, strongly represented pathways are presented as an interactome. A complete analysis is presented in Supplementary Data S3.

**Figure A4:**
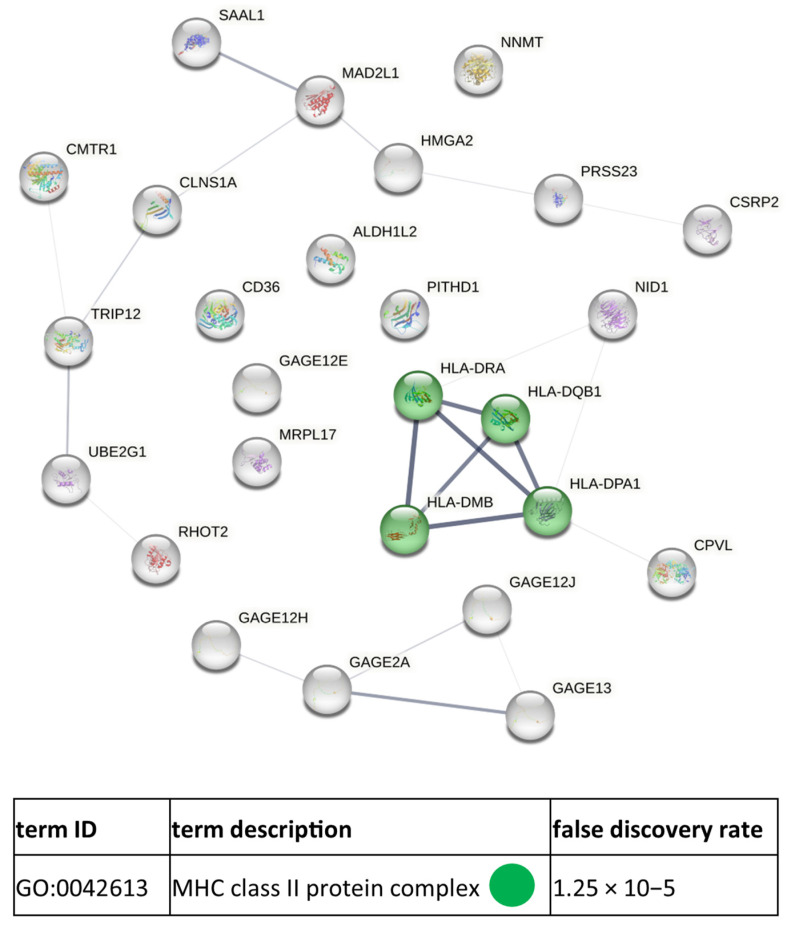
Diagram of functional protein association networks prepared with the use of STRING v. 11.0 software for proteins identified in WM115 cells but not found in any replicates of WM266-4 cells. The selected, strongly represented pathways are presented as an interactome. A complete analysis is presented in Supplementary Data S3.

In addition, the STRING v. 11.0 platform was used to prepare diagrams of functional protein association networks for proteins unique to given CM cell lines. The unique proteins from WM793 cells were shown to be enriched in, among others, the PPAR signaling pathway and vitamin D receptor pathway proteins (Figure A2). In the case of WM1205Lu cells, a unique protein enrichment in proteins with a Pleckstrin homology domain (PH domain) was observed (Figure A3). The PH domain is a protein domain found in many proteins involved in intracellular signaling or as components of the cytoskeleton. The unique proteins from WM115 cells were enriched in proteins related to the MHC class II protein complex responsible for presenting tumor antigens to the immune system (Figure A4). This corresponds to the primary character of WM115 cells, when the cells are not yet completely out of immune surveillance, as in the later stages of the disease. Finally, no enriched categories were found for the unique WM266-4 proteins.

**Figure A5:**
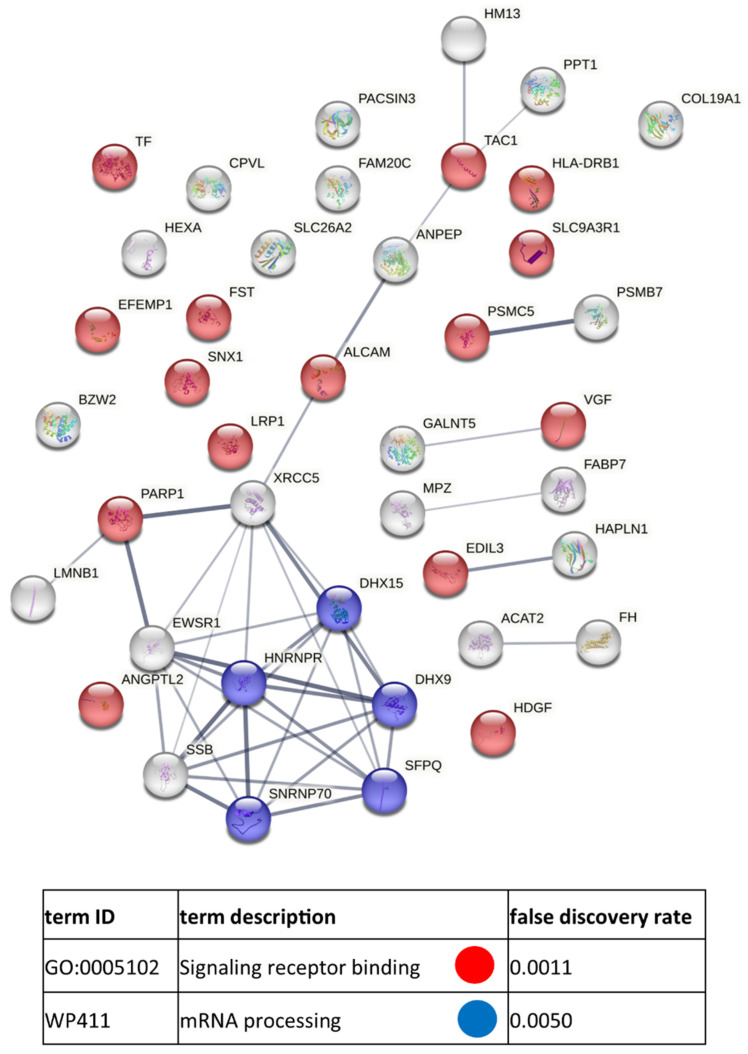
Diagram of functional protein association networks prepared with the use of STRING v. 11.0 software for proteins identified in WM793-derived exosomes cells but not found in any replicates of WM1205Lu exosomes. The selected, strongly represented pathways are presented as an interactome. A complete analysis is presented in Supplementary Data S3.

**Figure A6:**
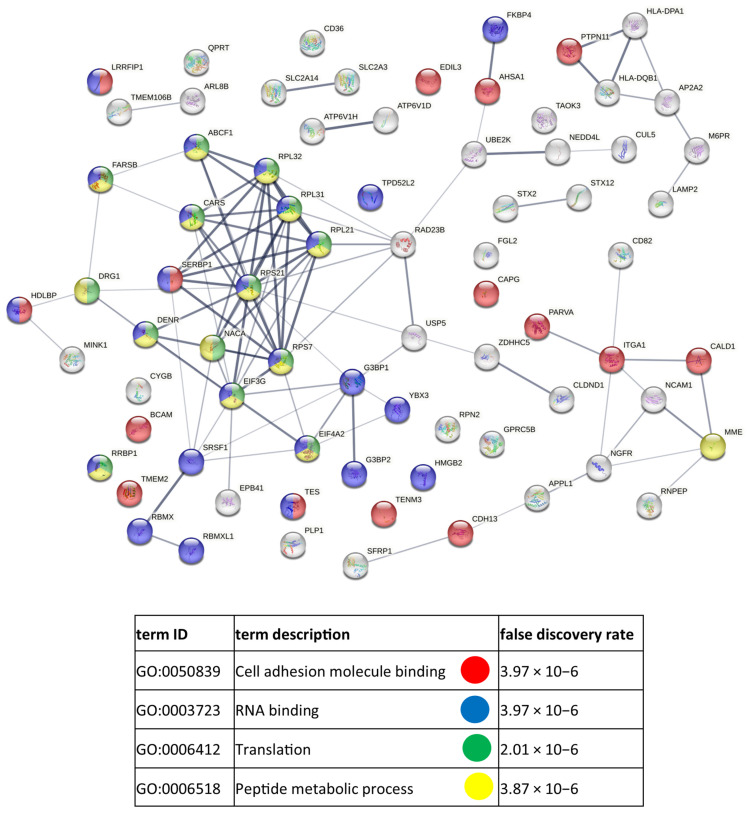
Diagram of functional protein association networks prepared with the use of STRING v. 11.0 software for proteins identified in WM115-derived exosomes but not found in any replicates of WM266-4-derived exosomes. The selected, strongly represented pathways are presented as an interactome. A complete analysis is presented in Supplementary Data S3.

**Figure A7:**
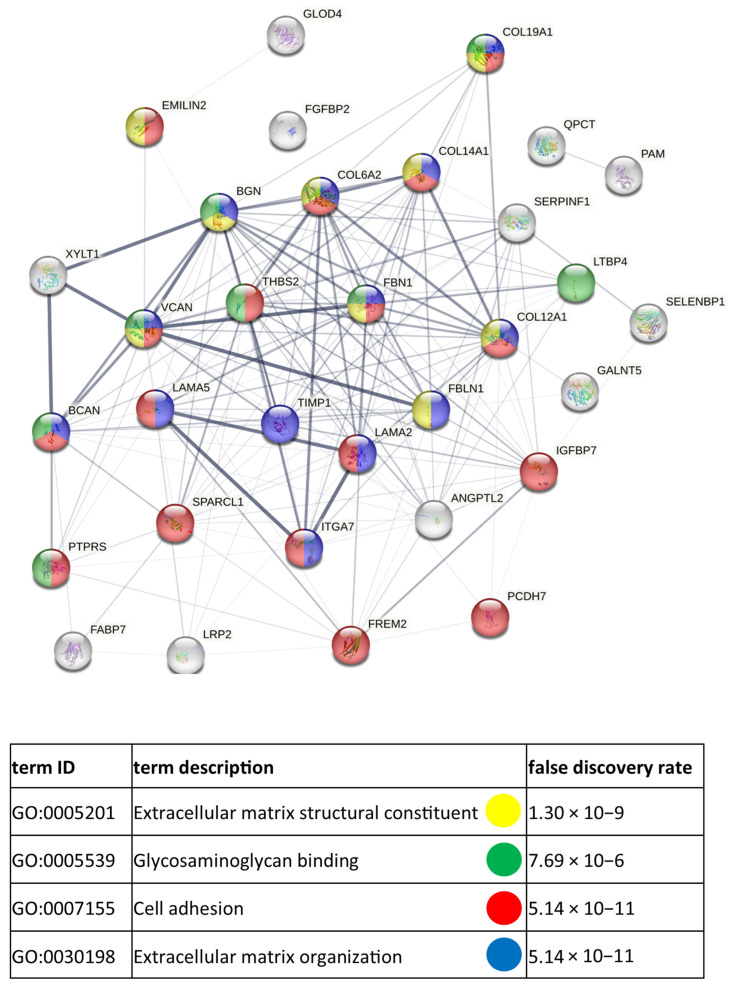
Diagram of functional protein association networks prepared with the use of STRING v. 11.0 software for proteins identified in WM266-4-derived exosomes but not found in any replicates of WM115-derived exosomes. The selected, strongly represented pathways are presented as an interactome. A complete analysis is presented in Supplementary Data S3.

Similar STRING diagrams were prepared for exosomal proteins. Proteins present in WM793-derived exosomes but absent in WM1205Lu-derived exosomes were enriched in proteins related to signaling receptor binding and mRNA processing (Figure A5). As only two proteins are unique for WM1205Lu, a similar analysis could not be performed. Furthermore, proteins identified in WM115-derived exosomes but absent in WM266-4-derived exosomes showed enrichment in categories related to cell adhesion molecule binding, RNA binding, translation, and peptide metabolic processes (Figure A6). For proteins identified in WM266-4-derived exosomes but absent in WM115-derived ones, similar generic categories were enriched, i.e., extracellular matrix structural constituent, extracellular matrix organization, glycosaminoglycan binding, and cell adhesion (Figure A7).

Label free quantification (LFQ) was also performed to determine differentially expressed proteins within isogenic pairs of CM cell lines and CM-derived exosomes. Proteins with fold change > 1.5 were considered upregulated in CM cells (*p* < 0.001), while for exosomes, a fold change of >1.2 and *p* < 0.05 were applied as threshold values. In summary, 243 proteins were upregulated in WM793 cells compared to WM1205Lu cells, and 185 were upregulated in WM1205Lu cells compared to WM793 cells. For the second isogenic pair of CM cell lines, WM115 cells contained 116 proteins that were upregulated compared to WM226-4 cells, while 160 proteins were found to be upregulated in WM266-4 compared to WM115 cells. Regarding exosomes, no differences in protein expression were observed between the proteins common to WM793-derived and WM1205Lu-derived exosomes. However, a total of 322 proteins were upregulated in WM115-derived exosomes compared to WM266-4-derived exosomes, while 77 proteins were upregulated in WM266-4-derived exosomes compared to WM115-derived exosomes. In summary, the ten proteins with the highest fold change for CM cell lines and exosomes are listed in Table 5, while the entire LFQ analysis is presented in Supplementary Data S3.

**Figure A8:**
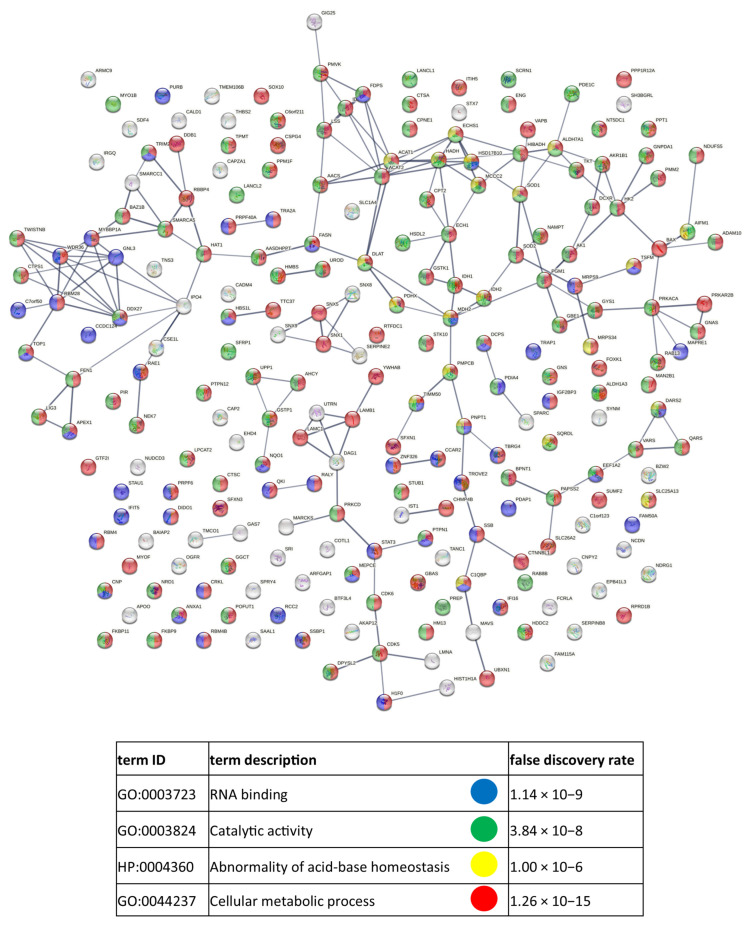
Diagram of functional protein association networks prepared with the use of STRING v. 11.0 software for proteins upregulated in WM793 cells vs. WM1205Lu cells. The selected, strongly represented pathways are presented as an interactome. Proteins with fold change > 1.5 were considered upregulated in WM793 cells (*p* < 0.001). A complete analysis is presented in Supplementary Data S3.

**Figure A9:**
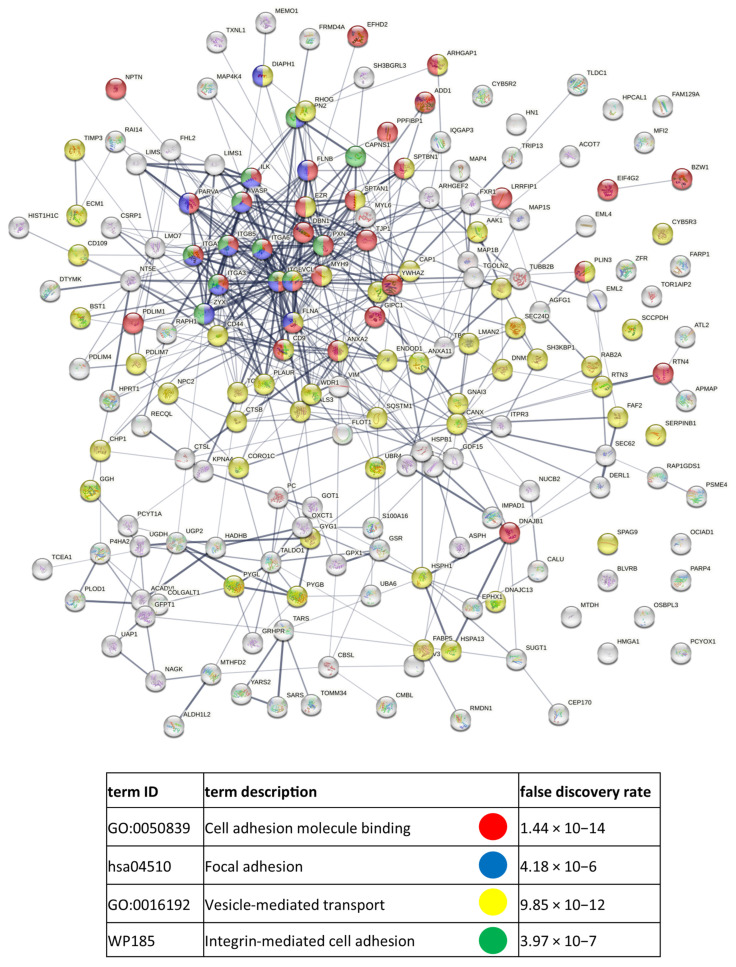
Diagram of functional protein association networks prepared with the use of STRING v. 11.0 software for proteins upregulated in WM1205Lu cells vs. WM793 cells. The selected, strongly represented pathways are presented as an interactome. Proteins with fold change > 1.5 were considered upregulated in WM1205Lu cells (*p* < 0.001). A complete analysis is presented in Supplementary Data S3.

**Figure A10:**
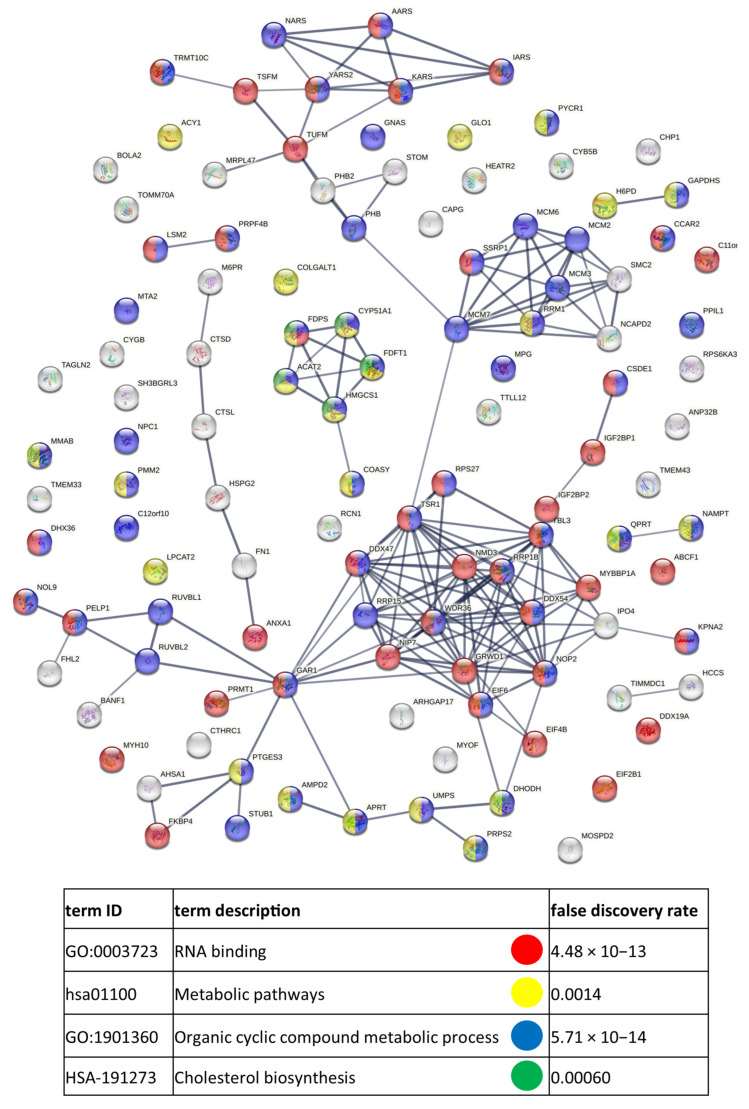
Diagram of functional protein association networks prepared with the use of STRING v. 11.0 software for proteins upregulated in WM115 cells vs. WM266-4 cells. The selected, strongly represented pathways are presented as an interactome. Proteins with fold change > 1.5 were considered upregulated in WM115 cells (*p* < 0.001). A complete analysis is presented in Supplementary Data S3.

**Figure A11:**
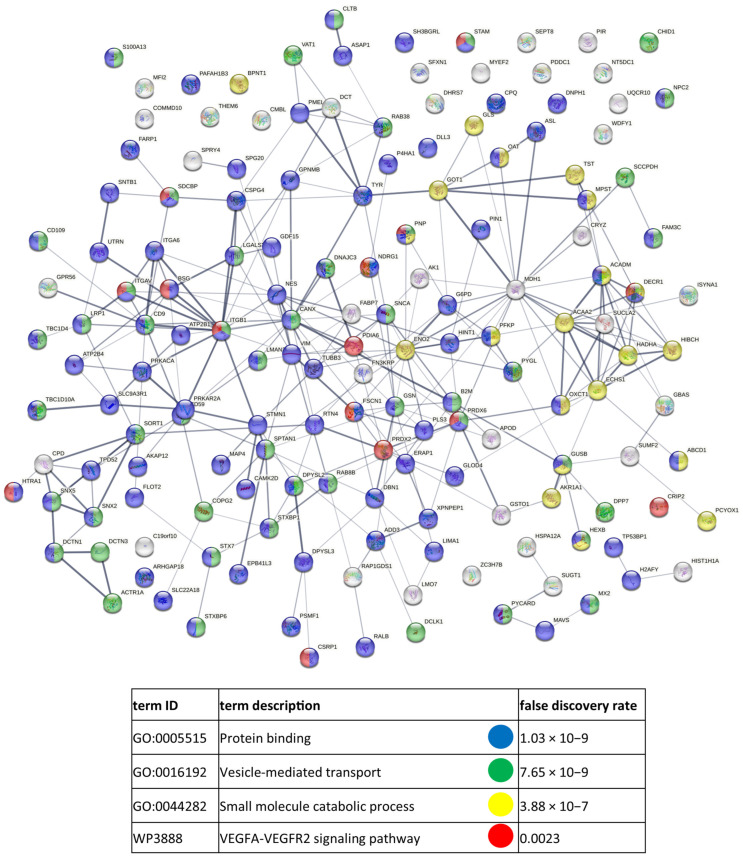
Diagram of functional protein association networks prepared with the use of STRING v. 11.0 software for proteins upregulated in WM266-4 cells vs. WM115 cells. The selected, strongly represented pathways are presented as an interactome. Proteins with fold change > 1.5 were considered upregulated in WM266-4 cells (*p* < 0.001). A complete analysis is presented in Supplementary Data S3.

**Figure A12:**
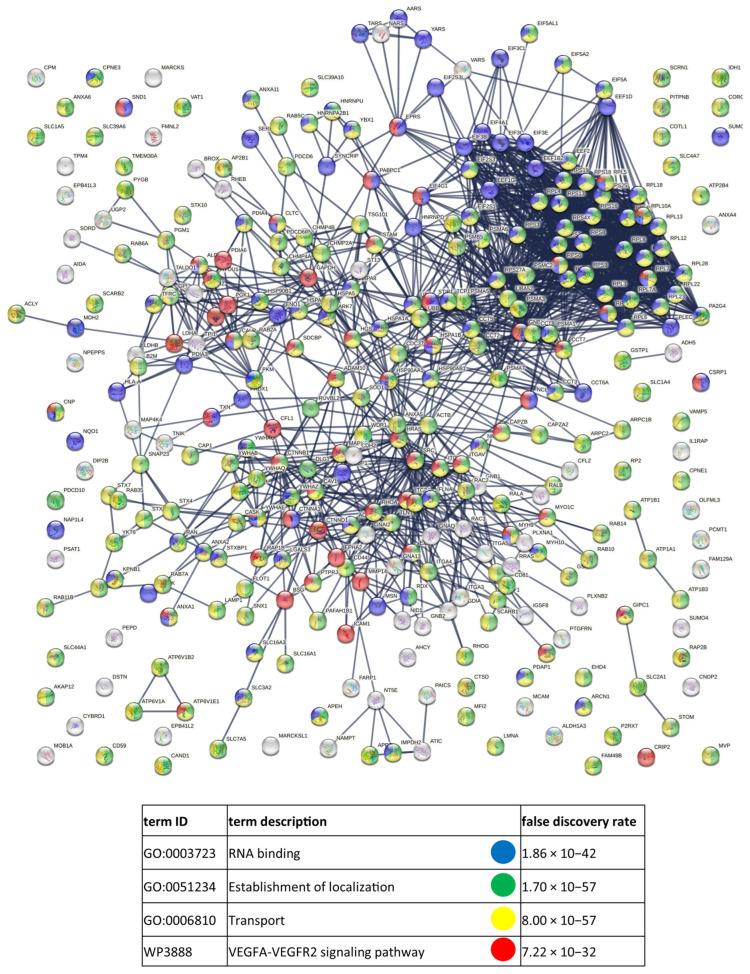
Diagram of functional protein association networks prepared with the use of STRING v. 11.0 software for proteins upregulated in WM115-derived exosomes vs. WM266-4-derived exosomes. The selected, strongly represented pathways are presented as an interactome. Proteins with fold change > 1.2 were considered upregulated in WM266-4 cells (*p* < 0.05). A complete analysis is presented in Supplementary Data S3.

**Figure A13:**
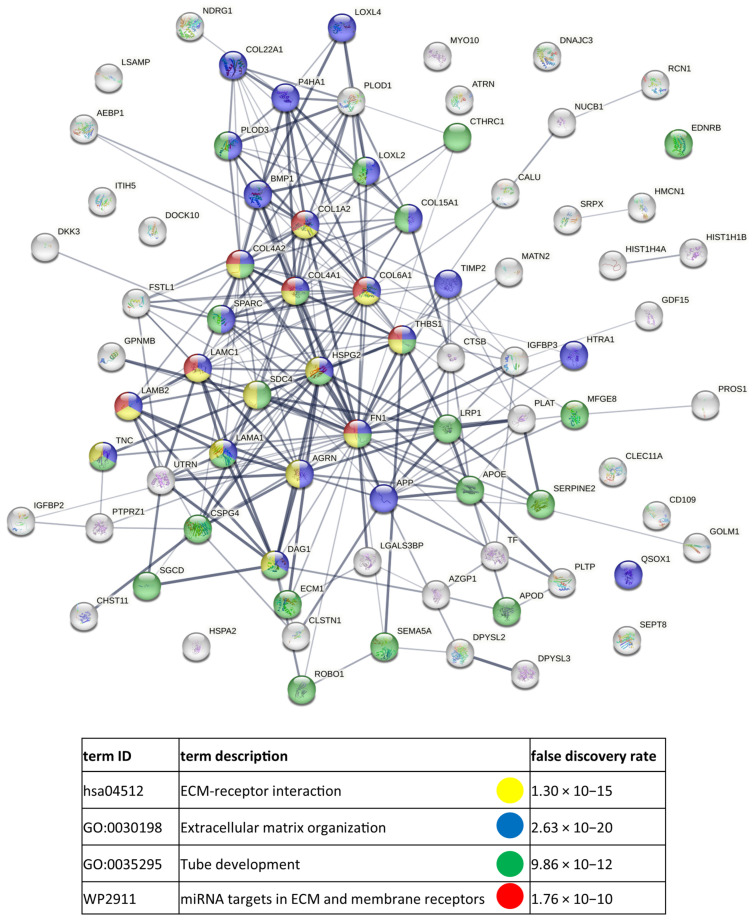
Diagram of functional protein association networks prepared with the use of STRING v. 11.0 software for proteins upregulated in WM266-4-derived exosomes vs. WM115-derived exosomes. The selected, strongly represented pathways are presented as an interactome. Proteins with fold change > 1.2 were considered upregulated in WM266-4 cells (*p* < 0.05). A complete analysis is presented in Supplementary Data S3.

The entire lists of upregulated proteins were later subjected to STRING analysis. For proteins upregulated in CM cell lines, the enriched categories included quite generic terms related to cell metabolism, cell adhesion, or RNA binding (Figure A8, Figure A9, Figure A10 and Figure A11). Interestingly, proteins upregulated in primary melanoma WM793 cells (compared to WM1205Lu cells) were enriched in the category of “abnormality of acid-base homeostasis”. This may be related to tumor acidosis, which often occurs within growing primary tumors until they develop a vascular network that increases the oxygen supply and restores normal pH levels. Regarding angiogenesis, proteins upregulated in WM266-4 cells were enriched in proteins involved in the vascular endothelial growth factor A/vascular endothelial growth factor receptor 2 (VEGFA-VEGFR2) signaling pathway. Signaling via VEGF induces the migration of endothelial cells during angiogenesis and may enhance microvascular permeability during tumor metastasis. Proangiogenic proteins were also upregulated in WM115-derived exosomes (category “VEGFA-VEGFR2 signaling pathway”) and WM266-4-derived exosomes (category “Tube development”) (Figure A12 and Figure A13). This suggests that exosomes released by primary tumor cells may be involved in proangiogenic signaling during the induction of angiogenesis. On the other hand, exosomes released by a metastatic tumor may contain proteins involved in the later stages of blood vessel formation such as sprouting or vessel maturation.

## 4. Discussion

### 4.1. CM-Derived Exosomes Are Enriched in Proteins with Functional Implications in Cancer

An increasing amount of evidence confirms that CM-derived exosomes increase the invasive potential of various recipient cells and drive the metastatic spread of melanoma tumors. An increased invasiveness after treatment with CM-derived exosomes was already demonstrated for melanocytes [25] as well as bone marrow-derived stromal cells [26]. Consequently, in the present study, CM-derived exosomes were more enriched in proteins involved in positive regulation of cell migration than CM cells (Figure 8) based on GO analysis. It suggests that proteins with a promigratory function are preferentially sorted into exosomes by CM cells.

Spreading of CM cells is connected to the activity of matrix metalloproteinases (MMPs), and positive correlation between the concentration of exosomes, the number of lytic enzymes in their cargo, and their pro-invasive capabilities have been demonstrated [27,28]. CM-derived EVs have already been shown to carry several MMPs and their endogenous activator CD147 [29,30,31,32,33]. In the present study, MMP1 was identified only in WM793 cells, while MMP14 was found in WM266-4 cells and all exosome samples except WM1205Lu-derived exosomes. In contrast, CD147 and other tissue inhibitors of MMPs (TIMP1, TIMP2, TIMP3) were present in all exosome samples, suggesting that CM exosomes may not always transfer MMPs but that they have a regulatory role towards MMP activity.

Regarding proteins involved in CM metastasis, c-Met and Rab27a proteins were shown to be transferred via exosomes [34,35], and the knockdown of Rab27a decreased invasiveness and metastasis of CM cells as well as exosome secretion [34]. A more recent proteomic study revealed enrichment of CM-derived exosomes in proteins belonging to the prometastatic NRAS, SRC, KIT, EGFR, and MET signaling pathways [36]. In the present study, Rab27 was identified in WM115 and WM266-4 cells, but it did not appear in exosome samples. On the other hand, Src kinase, NRAS GTPase, and the epidermal growth factor receptor (EGFR) were identified in all exosome samples, but only Src kinase was identified in CM cells. The abundance of NRAS and EGFR in CM cells may not be high enough for them to be identified among more abundant cellular proteins. Furthermore, NRAS and EGFR are likely to be enriched in CM-derived exosomes, demonstrating their role in providing key players in prometastatic signaling pathways.

In addition, there is substantial evidence that CM-derived exosomes may contribute to organotropisms during CM metastatis. Several in vivo studies have reported the involvement of CM exosomes in the organ-specific formation of secondary tumors in the lungs [37], bone [38], and sentinel lymph nodes [39]. CM-derived exosomes may also contribute to the formation of brain metastasis. Using a biomimetic blood-brain barrier (BBB) model (co-culture of brain microvascular endothelial cells, astrocytes, and microglial cells), CM-derived exosomes have been shown to induce endothelial damage, disrupt BBB integrity, and induce glial activation [40]. Finally, CM-derived exosomes upregulated proteins from the MAPK signaling pathway in primary melanocytes to induce epithelial-mesenchymal transition (EMT) and promote the metastatic phenotype [41]. In the present study, MAPK1, MAPK2, MAPK3, and MAPK14 were identified in CM cells, while MAPK1 and MAP4K4 were present in most of the CM-derived exosome samples.

Exosomes also participate in the delivery of proangiogenic factors or increase their expression in recipient cells. A study by Hood et al. showed that CM-derived exosomes induce the formation of endothelial spheroids [42]. Another study showed that CM cells release exosomes enriched in interleukin 8 (IL-8), vascular endothelial growth factor (VEGF), MMP2, and IL-6 [43]. CM-derived exosomes containing the urokinase plasminogen activator receptor (uPAR) were shown to promote angiogenesis in recipient endothelial cells by upregulating VE-Cadherin, EGFR, and uPAR expression, and enhancing ERK1,2 signaling [44]. Alternatively, activation of the JAK-STAT pathway and enhanced angiogenesis were observed in endothelial cells after treatment with CM-derived exosomes [45]. In the present study, CM-derived exosomes were more enriched in proteins involved in angiogenesis than CM cells (Figure 8), including proteins from the VEGF signaling pathway. However, no VEGF was found in any sample. Instead, other proangiogenic factors were identified in both CM cells and CM-derived exosomes such as neuropilin 1, annexin A2, or integrin subunits (α5, αV).

CM-derived exosomes can also induce lymphangiogenesis and thus contribute to lymph-node metastasis [46,47]. Potential mechanisms include ERK kinase induction, nuclear factor (NF)-κB activation, and increased expression of intracellular adhesion molecule (ICAM)-1 expression in lymphatic endothelial cells [47]. Importantly, it has been shown that exosomal transfer of the nerve growth factor receptor is responsible for the aforementioned effects. In the present study, NGFR was identified in WM115 exosomes, supporting the hypothesis that primary melanoma tumors may release NGFR-containing exosomes to enhance formation of lymph-node metastasis.

In addition to endothelial cells, exosomes can modulate the function of other cells present in the tumor microenvironment such as fibroblasts and immune cells. In a recent study, cancer-associated fibroblasts were activated by the CM-derived exosomes to a greater degree than normal fibroblasts for the transcription of genes for proinflammatory cytokines and chemokines, mainly IL-6 or IL-8 [48]. CM exosomes can also induce proinflammatory polarization and activation of macrophages [49,50]. They can also impair the function [51,52] or induce apoptosis [46] of CD8+ cytotoxic T-cells to locally suppress anti-tumor cytotoxicity. On the other hand, CM exosomes can directly activate CD4+ helper T-cells through the transfer of miR690 and Rab27a [53]. Finally, CM exosomes were shown to reduce the differentiation of bone marrow-derived dendritic cells [54]. In the present study, multiple GO categories related to immune response were enriched for CM cells and CM-derived exosomes. Specifically, CM exosomes were enriched in proteins involved in leukocyte and neutrophil migration (Figure 8). This suggests the involvement of CM-derived exosomes in either enhancing or inhibiting the influx of immune cells into melanoma tumors and the subsequent immune response.

### 4.2. Clinical Relevance of Proteins Identified in CM-Derived Exosomes

It has been shown that the number and/or composition of exosome proteins changes when the parental cell undergoes neoplastic transformation. Cancer cells are known to secrete more exosomes than normal cells, so there is an interest in using exosome concentration as a biomarker. A significantly higher concentration of released exosomes was observed for CM cells compared to normal melanocytes [25], and for metastatic CM cells compared to primary CM cells [55]. However, in the present study, NTA showed a rather similar concentration of exosomes in samples from four CM cell lines (approx. 5 × 10^7^ particles/mL) after taking equal volumes of conditioned media for exosome isolation. This suggests that sole exosome concentration may not be a good indicator of disease stage, as opposed to being a marker of disease occurrence.

Currently, lactate dehydrogenase A (LDH) or S100 calcium binding protein B (S100B) are the two most important clinical markers of CM. Serum levels of S100B increase with tumor growth and are used to monitor patients in advanced stages of the disease [56,57]. In the present study, S100B was identified in all CM cell samples and in exosomes derived from WM793 and WM115 cells. The lack of S100B in exosomes derived from metastatic WM1205Lu and WM266-4 cells may be potentially useful in discriminating between primary and metastatic CM. Interestingly, in our previous study, S100B was present in ectosomes from all CM cell lines, regardless of disease stage [33]. This suggests that the diagnostic target for S100B protein should not be ectosomes but rather exosomes. Finally, all CM cell and CM exosome samples contained LDH, which is now being used in clinical practice as a predictor of CM patient survival [58].

Recently, EV-oriented studies have moved forward in the CM biomarker field. First, the levels of MET, dopachrome tautomerase (TYRP2), integrin α4β1 (VLA-4), Hsp-90, and Hsp-70 were found to be upregulated in plasma-derived exosomes of CM patients compared to healthy controls [34]. In the present study, Hsp-90, Hsp-70, and α4 integrin subunits were identified in all samples, while TYRP2 was found only in WM115 and WM266-4 cells. Nevertheless, none of these proteins displayed differential expression based on LFQ; therefore, their biomarker potential needs further evaluation. Other studies have shown increased levels of CD63 tetraspanin in exosomes from both CM cell cultures (compared to normal cells) [59] and plasma of CM patients (compared to healthy controls) [60]. However, in the present study, CD63 expression was not significantly different between samples. Exosomes can also be isolated from less obvious sources, such as fluid from lymphatic drainage of melanoma tumors. It was shown that such exosomes are enriched in B-raf protein with V600E mutation, and its level was correlated with the risk of disease recurrence [61]. Here, B-raf protein was identified in samples from all CM cells, but it was not detected in CM-derived exosomes, possibly due to the source of isolated EVs.

Exosomal proteins are also potential biomarkers of treatment response. In melanoma patients responding positively to treatment with nivolumab and pembrolizumab (combination of antibodies against programmed cell death protein 1/programmed cell death protein ligand 1 (PD1/PD-L1)), a significant decrease in exosomal PD-L1 expression was observed [62,63]. In the present study, neither PD1 nor PD-L1 was identified in CM cells and exosomes. However, several immunosuppressive proteins from the family of programmed cell death protein were identified in CM cells, such as PD3, PD5, PD6, PD10, PD11, and PD4—a potent inhibitor of neoplastic transformation. On the other hand, CM-derived exosomes (besides the WM115-derived sample) contained only PD6 and PD10, suggesting that this group of proteins is not preferentially carried by exosomes. In addition, exosomal CD73 ectonucleotidase (producing adenosine—suppressor of T-cell function) has been found to increase in CM patients unresponsive to treatment with anti-PD-1 agents [64]. In addition, Pietrowska et al. identified 75 proteins upregulated in plasma-derived exosomes from CM patients with progressive disease compared with patients showing no evidence of CM after therapy. Programmed cell death 6-interacting protein (PDCD6IP) showed the highest upregulation in exosomes from patients with disease progression, while contactin-1 (CNTN1) was upregulated in exosomes from patients in remission [65]. CD73 and PDCD6IP were also present in all exosome samples in the present study, demonstrating their potential as a treatment response biomarker in CM.

Further biomarker targets were selected by meta-analysis of exosome-enriched proteins from CM cells based on the Prognoscan database [55]. The study identified 11 targets significantly correlated with metastasis and poor prognosis [55]. Seven of these 11 proteins—i.e., GTPase HRas (HRAS), neutral alpha-glucosidase AB (GANAB), cofilin-2 (CFL2), Hsp90B1, hypoxia upregulated protein 1 (HYOU1), gelsolin (GSN), and TIMP3—were identified in all CM cells and exosome samples in the present study. Interestingly, GTPase NRas (NRAS) and HspA5 were only found in exosome samples, and their enrichment in CM-derived exosomes vs. cells should become the subject of further investigation. Regarding LFQ analysis (Table 5), it was found that GSN was upregulated in WM266-4 cells compared to WM115 cells, while TIMP3 was upregulated in WM1205Lu cells vs. WM793 cells. On the other hand, HRAS, CFL2, Hsp90B1, Hsp90AB1, HYOU1, and HspA5 were all upregulated in WM115-derived exosomes compared to WM266-4-derived exosomes.

Finally, a qualitative and quantitative comparison of CM cells and CM-derived exosomes in isogenic pairs of cell lines (WM793 vs. WM1205Lu and WM115 vs. WM266-4) revealed many more potential targets for protein biomarkers than mentioned in Section 4. This includes unique proteins (Table 1, Table 2, Table 3 and Table 4) or proteins with differential expression (Table 5). Focusing on CM-derived exosomes, differentially expressed proteins were identified only between WM115-derived and WM266-4-derived exosomes. Among proteins with the greatest fold change (Table 5), there are several with well-known implications in melanoma progression. For example, annexin A1 (upregulated in WM115-derived exosomes) is a known promoter of primary melanoma tumor dissemination, mainly by inhibiting E-cadherin expression [66,67]. Additionally, A-kinase anchor protein 12 (upregulated in WM115-derived exosomes) shifts PKA-mediated protein phosphorylation to increase CM cell migration and metastasis [68]. Regarding proteins upregulated in WM266-4-derived exosomes, tenascin mediates protective signals in therapy-resistant melanomas by downregulation of multiple ATP-binding cassette transporters [69]. Therapeutically, upregulation of adipocyte enhancer-binding protein 1 (also upregulated in WM-266-4-derived exosomes) has also been shown to contribute to resistance to BRAF (V600E) inhibition in the treatment of melanoma [70]. Finally, semaphorin 5A is known to regulate melanoma cell migration and invasion, and angiogenesis [71,72].

Nevertheless, larger preclinical and clinical trials are required to further validate any novel CM biomarker identified in exosomes in vitro. The clinical potential of exosomes is based on the development of efficient isolation protocols. Such protocols must provide highly concentrated, uncontaminated EV samples, optimally preserving the native form and function. Based on the promising results from the present study, the search for biomarkers for CM should continue. Similar high-throughput proteomic approaches could help overcome the current lack of effective diagnostic and prognostic tools.

## 5. Conclusions

Despite advances in diagnostics and treatment, patients with CM still face a poor prognosis. Specific alterations to the proteomic profiles of CM cell lines representing different stages of disease and exosomes derived from those cell lines were reported in the present study. In addition to these unique features of CM cells and exosomes, a conserved part of their proteome was also described, which can be used by various tumor cells to promote their growth and dissemination. Our description of the complex composition of cellular and exosomal proteins and their related functions provides deeper insight into the role of exosomes in CM progression.

Our results also point to some of the exosomal proteins to be evaluated as potential circulating CM biomarkers. This includes the most commonly used CM markers such as LDH and S100B. Additionally, all unique proteins (Table 3 and Table 4) or proteins with the most differential expression between exosomes from primary and metastatic CM cells (Table 5)—such as annexin A1 [66,67], A-kinase anchor protein 12 [68], tenascin [69], adipocyte enhancer-binding protein 1 [70], and semaphorin 5A [71,72]—should be evaluated. In addition, some proteins with biomarker potential assessed by other groups were also detected in CM exosomes in the present study. This includes proteins with diagnostic potential (α4β1 integrin, Hsp-90, and Hsp-70 [34]) or those that can discriminate patients at different stages (GTPase HRas, cofilin-2, hypoxia upregulated protein 1, Hsp90B1, Hsp90AB1, and HspA5 [55]). Finally, exosomal CD73 [64] and PDCD6IP [65] appear to be promising potential treatment response biomarkers in CM.

## Figures and Tables

**Table 1:** List of proteins identified in WM793 cells but not found in any replicates of WM1205Lu cell samples and vice versa.

Present Only in WM793 Cells		Present Only in WM1205Lu Cells
Structural maintenance of chromosomes flexible hinge domain-containing protein 1 Procollagen-lysine,2-oxoglutarate 5-dioxygenase 2 Acyl-CoA desaturase Interferon-induced protein with tetratricopeptide repeats 3 Plexin-B2 Fatty acid-binding protein, brain mRNA cap guanine-N7 methyltransferase Syntaxin-6 TBC1 domain family member 4 Poly(A)-specific ribonuclease PARN E3 ubiquitin-protein ligase HERC2 Adenosine deaminase HLA class II histocompatibility antigen, DR alpha chain HLA class II histocompatibility antigen, DRB1-15 beta chain Interstitial collagenase 22 kDa interstitial collagenase 27 kDa interstitial collagenase Plasminogen activator inhibitor 2 Ubiquitin-like protein ISG15 Acyl-CoA-binding protein Complement decay-accelerating factor Interferon-induced protein with tetratricopeptide repeats 1 Proto-oncogene tyrosine-protein kinase Src Histone H1.5 Plasma membrane calcium-transporting ATPase 1 Erythrocyte band 7 integral membrane protein Delta-1-pyrroline-5-carboxylate dehydrogenase, mitochondrial Serine hydroxymethyltransferase, cytosolic Alpha-synuclein Lysosomal acid lipase/cholesteryl ester hydrolase Cyclin-dependent kinase inhibitor 2A Cyclin-dependent kinase 4 inhibitor B Cytosolic phospholipase A2 Phospholipase A2 Lysophospholipase Glutamate dehydrogenase 2, mitochondrial Signal transducer and activator of transcription 2 Integrin alpha-1 Guanine nucleotide-binding protein G(i) subunit alpha-1 HLA class II histocompatibility antigen, DR beta 3 chain	Hydroxymethylglutaryl-CoA synthase, cytoplasmic Semaphorin-3B Nuclear factor of activated T-cells, cytoplasmic 2 Caspase-8 Caspase-8 subunit p18 Caspase-8 subunit p10 Transmembrane glycoprotein NMB 3-beta-hydroxysteroid-Delta(8),Delta(7)-isomerase Nicotinate-nucleotide pyrophosphorylase [carboxylating] Kynureninase Acyl-coenzyme A thioesterase THEM4 ERO1-like protein beta Extracellular serine/threonine protein kinase FAM20C Kelch repeat and BTB domain-containing protein 2 Phosphatidylinositol 3,4,5-trisphosphate-dependent Rac exchanger 1 protein PH-interacting protein Ubiquitin-conjugating enzyme E2 E3 Molybdenum cofactor sulfurase Zinc finger CCCH-type antiviral protein 1-like Protein FAM84B Methylcrotonoyl-CoA carboxylase subunit alpha, mitochondrial Protein NipSnap homolog 1 O-acetyl-ADP-ribose deacetylase MACROD1 Solute carrier family 12 member 9 Protein tweety homolog 3 Egl nine homolog 1 Activity-dependent neuroprotector homeobox protein Probable serine carboxypeptidase CPVL DNA-directed RNA polymerase I subunit RPA2 ATP-binding cassette sub-family B member 6, mitochondrial Exosome complex component RRP41 Leucine zipper transcription factor-like protein 1 Ethylmalonyl-CoA decarboxylase Leucine-rich repeat and WD repeat-containing protein 1 LIM domain-containing protein 1 Protein NDRG3 Protein kinase C and casein kinase substrate in neurons protein 3 Collagen type IV alpha-3-binding protein 28S ribosomal protein S35, mitochondrial	Citron Rho-interacting kinase Neuropilin-1 Triple functional domain protein Galactosylgalactosylxylosylprotein 3-beta-glucuronosyltransferase 3 Glutamine--fructose-6-phosphate aminotransferase [isomerizing] 2 Sorbin and SH3 domain-containing protein 2 Tissue-type plasminogen activator;Tissue-type plasminogen activator chain A;Tissue-type plasminogen activator chain B Alpha-crystallin B chain Apolipoprotein D Neuromodulin Protein S100-A1 Thromboxane-A synthase CAP-Gly domain-containing linker protein 1 Carbonic anhydrase-related protein AP-1 complex subunit sigma-2 TNF receptor-associated factor 1 Growth factor receptor-bound protein 10 Calcyphosin Dihydropyrimidinase-related protein 1 Arf-GAP with coiled-coil, ANK repeat and PH domain-containing protein 2 Glycerol-3-phosphate acyltransferase 3 HCLS1-binding protein 3 Uncharacterized protein FLJ45252 Fermitin family homolog 3 5-nucleotidase domain-containing protein 3 Protein AHNAK2 Actin filament-associated protein 1-like 2 EH domain-binding protein 1 E3 ubiquitin-protein ligase DTX3L PDZ domain-containing protein GIPC3 Ubiquitin carboxyl-terminal hydrolase 47 Sorting nexin-18 Protein FAM107B Synaptic vesicle membrane protein VAT-1 homolog-like T-complex protein 11-like protein 1 EH domain-containing protein 2 Arf-GAP with SH3 domain, ANK repeat and PH domain-containing protein 1 Absent in melanoma 1 protein

**Table 2:** List of proteins identified in WM115 cells but not found in any replicates of WM266-4 cell samples and vice versa.

Present Only in WM115 Cells	Present Only in WM266-4 Cells	
Serine protease 23 HLA class II histocompatibility antigen, DR alpha chain HLA class II histocompatibility antigen, DQ beta 1 chain HLA class II histocompatibility antigen, DR beta 4 chain Nidogen-1 Platelet glycoprotein 4 HLA class II histocompatibility antigen, DP alpha 1 chain HLA class II histocompatibility antigen, DM beta chain Nicotinamide N-methyltransferase High mobility group protein HMGI-C Methylosome subunit pICln Ubiquitin-conjugating enzyme E2 G1 Ubiquitin-conjugating enzyme E2 G1, N-terminally processed HLA class II histocompatibility antigen, DR beta 3 chain Mitotic spindle assembly checkpoint protein MAD2A E3 ubiquitin-protein ligase TRIP12 Cysteine and glycine-rich protein 2 Mitochondrial 10-formyltetrahydrofolate dehydrogenase G antigen 13 G antigen 2D G antigen 2A G antigen 2B/2C G antigen 12J G antigen 12H G antigen 12B/C/D/E Mitochondrial Rho GTPase 2 Cap-specific mRNA (nucleoside-2-O-)-methyltransferase 1 Protein SAAL1 PITH domain-containing protein 1 Probable serine carboxypeptidase CPVL 39S ribosomal protein L17, mitochondrial	Shootin-1 Monocarboxylate transporter 4 Syndecan-3 Heat shock 70 kDa protein 4L Cholinesterase Annexin A3 Insulin-like growth factor-binding protein 2 Protein S100-A1 Caspase-1 Caspase-1 subunit p20 Caspase-1 subunit p10 Neural cell adhesion molecule L1 Sulfotransferase 1A1 Sulfotransferase 1A2 H(+)/Cl(−) exchange transporter 3 Serine beta-lactamase-like protein LACTB, mitochondrial Collagen alpha-1 (XIV) chain Selenium-binding protein 1 Inactive tyrosine-protein kinase 7 AP2-associated protein kinase 1 NADH-cytochrome b5 reductase 2 Nucleoredoxin Uncharacterized protein FLJ45252 Myosin-14 MICAL-like protein 1 Probable aminopeptidase NPEPL1 PRKC apoptosis WT1 regulator protein Ras-related protein Rab-34 Leucine-rich repeat-containing protein C10orf11 Pleckstrin homology domain-containing family A member 2 Ras-related protein Rab-18 Coatomer subunit zeta-2 Calcium-dependent secretion activator 1 Integral membrane protein 2B BRI2, membrane form BRI2 intracellular domain BRI2C, soluble form	Bri23 peptide FH1/FH2 domain-containing protein 1 Sulfide:quinone oxidoreductase, mitochondrial

**Table 3:** List of proteins identified in WM793-derived exosomes but not found in any replicates of WM1205Lu-derived exosomes and vice versa.

Present Only in WM793 Exosomes		Present Only in WM1205Lu Exosomes
Na(+)/H(+) exchange regulatory cofactor NHE-RF1 Neurosecretory protein VGF Neuroendocrine regulatory peptide-1 Neuroendocrine regulatory peptide-2 Antimicrobial peptide VGF [554–577] Fatty acid-binding protein, brain Pre-mRNA-splicing factor ATP-dependent RNA helicase DHX15 Heterogeneous nuclear ribonucleoprotein R EGF-like repeat and discoidin I-like domain-containing protein 3 HLA class II histocompatibility antigen, DRB1-15 beta chain Serotransferrin Lupus La protein Beta-hexosaminidase subunit alpha Fumarate hydratase, mitochondrial U1 small nuclear ribonucleoprotein 70 kDa Heterogeneous nuclear ribonucleoprotein A1 Heterogeneous nuclear ribonucleoprotein A1, N-terminally processed Heterogeneous nuclear ribonucleoprotein A1-like 2 Poly [ADP-ribose] polymerase 1 Hyaluronan and proteoglycan link protein 1 X-ray repair cross-complementing protein 5 Aminopeptidase N Follistatin Protachykinin-1 Substance P Neurokinin A Neuropeptide K Neuropeptide gamma C-terminal-flanking peptide Lamin-B1 Splicing factor, proline- and glutamine-rich Myelin protein P0	Sulfate transporter Palmitoyl-protein thioesterase 1 Hepatoma-derived growth factor 26S protease regulatory subunit 8 HLA class II histocompatibility antigen, DR beta 3 chain Protein SET; Protein SETSIP RNA-binding protein EWS Prolow-density lipoprotein receptor-related protein 1 Low-density lipoprotein receptor-related protein 1 85 kDa subunit Low-density lipoprotein receptor-related protein 1 515 kDa subunit Low-density lipoprotein receptor-related protein 1 intracellular domain ATP-dependent RNA helicase A EGF-containing fibulin-like extracellular matrix protein 1 Sorting nexin-1 CD166 antigen Collagen alpha-1(XIX) chain Polypeptide N-acetylgalactosaminyltransferase 5 Extracellular serine/threonine protein kinase FAM20C Minor histocompatibility antigen H13 Proteasome subunit beta type-7 Acetyl-CoA acetyltransferase, cytosolic Probable serine carboxypeptidase CPVL Protein kinase C and casein kinase substrate in neurons protein 3 Angiopoietin-related protein 2 Basic leucine zipper and W2 domain-containing protein 2	Fermitin family homolog 3 Ras GTPase-activating-like protein IQGAP3

**Table 4:** List of proteins identified in WM115-derived exosomes but not found in any replicates of WM266-4-derived exosomes and vice versa.

Present Only in WM115 Exosomes			Present Only in WM266-4 Exosomes
Nascent polypeptide-associated complex subunit alpha, muscle-specific form Nascent polypeptide-associated complex subunit alpha Tumor protein D54 Density-regulated protein EGF-like repeat and discoidin I-like domain-containing protein 3 Eukaryotic translation initiation factor 3 subunit G AP-2 complex subunit alpha-2 Activator of 90 kDa heat shock protein ATPase homolog 1 HLA class II histocompatibility antigen, DQ beta 1 chain Dolichyl-diphosphooligosaccharide--protein glycosyltransferase subunit 2 Tumor necrosis factor receptor superfamily member 16 Neprilysin Solute carrier family 2, facilitated glucose transporter member 3 Solute carrier family 2, facilitated glucose transporter member 14 Protein 4.1 Lysosome-associated membrane glycoprotein 2 Neural cell adhesion molecule 1 HLA class II histocompatibility antigen, DR beta 4 chain Platelet glycoprotein 4 Y-box-binding protein 3 HLA class II histocompatibility antigen, DP alpha 1 chain Cation-dependent mannose-6-phosphate receptor High mobility group protein B2	Syntaxin-2 RNA-binding motif protein, X chromosome RNA-binding motif protein, X chromosome, N-terminally processed RNA binding motif protein, X-linked-like-1 Macrophage-capping protein Ubiquitin carboxyl-terminal hydrolase 5 60S ribosomal protein L21 Cysteine--tRNA ligase, cytoplasmic Basal cell adhesion molecule UV excision repair protein RAD23 homolog B Cadherin-13 Integrin alpha-1 Myelin proteolipid protein Ubiquitin-conjugating enzyme E2 K 40S ribosomal protein S7 60S ribosomal protein L31 60S ribosomal protein L32 40S ribosomal protein S21 Vigilin Peptidyl-prolyl cis-trans isomerase FKBP4 Peptidyl-prolyl cis-trans isomerase FKBP4, N-terminally processed Caldesmon Tyrosine-protein phosphatase non-receptor type 11 Serine/arginine-rich splicing factor 1 Ras GTPase-activating protein-binding protein 1 Eukaryotic initiation factor 4A-II;Eukaryotic initiation factor 4A-II, N-terminally processed Fibroleukin	Nicotinate-nucleotide pyrophosphorylase [carboxylating] Leucine-rich repeat flightless-interacting protein 1 Syntaxin-12 Secreted frizzled-related protein 1 Misshapen-like kinase 1 Plasminogen activator inhibitor 1 RNA-binding protein ATP-binding cassette sub-family F member 1 Cytoglobin Cullin-5 E3 ubiquitin-protein ligase NEDD4-like Palmitoyltransferase ZDHHC5 Serine/threonine-protein kinase TAO3 Aminopeptidase B Phenylalanine--tRNA ligase beta subunit Transmembrane protein 106B Alpha-parvin ADP-ribosylation factor-like protein 8B Claudin domain-containing protein 1 G-protein coupled receptor family C group 5 member B Teneurin-3 Ribosome-binding protein 1 Testin Transmembrane protein 2 V-type proton ATPase subunit H DCC-interacting protein 13-alpha Ras GTPase-activating protein-binding protein 2 Developmentally-regulated GTP-binding protein 1 V-type proton ATPase subunit D Nicotinate-nucleotide CD82 antigen	Laminin subunit alpha-5 Fatty acid-binding protein, brain Protocadherin-7 Metalloproteinase inhibitor 1 Collagen alpha-2(VI) chain Versican core protein Peptidyl-glycine alpha-amidating monooxygenase;Peptidylglycine alpha-hydroxylating monooxygenase Peptidyl-alpha-hydroxyglycine alpha-amidating lyase Biglycan Fibulin-1 Laminin subunit alpha-2 Thrombospondin-2 Fibrillin-1 Pigment epithelium-derived factor Low-density lipoprotein receptor-related protein 2 Collagen alpha-1(XIV) chain Selenium-binding protein 1 Receptor-type tyrosine-protein phosphatase S Integrin alpha-7;Integrin alpha-7 heavy chain Integrin alpha-7 light chain;Integrin alpha-7 70 kDa form SPARC-like protein 1 Collagen alpha-1(XIX) chain Insulin-like growth factor-binding protein 7 Glutaminyl-peptide cyclotransferase FRAS1-related extracellular matrix protein 2 Polypeptide N-acetylgalactosaminyltransferase 5 Xylosyltransferase 1 Latent-transforming growth factor beta-binding protein 4 Brevican core protein Collagen alpha-1(XII) chain EMILIN-2 Fibroblast growth factor-binding protein 2 Glyoxalase domain-containing protein 4 Angiopoietin-related protein 2

**Table 5:** Differentially expressed proteins within isogenic pairs of CM cells lines and CM-derived exosomes. Ten proteins with the highest fold change for CM cell lines and exosomes are listed in this table, while the entire LFQ analysis is presented in Supplementary Data.

**Upregulated in WM793 Cells**		**Upregulated in WM1205Lu Cells**	
**Proteins**	**Fold Change**	**Proteins**	**Fold Change**
Unconventional myosin-Ib	16.750	UDP-N-acetylhexosamine pyrophosphorylase	22.502
Sulfate transporter	7.4312	Tight junction protein ZO-1	19.663
Inter-alpha-trypsin inhibitor heavy chain H5	7.259	Protein Niban	18.700
Death-inducer obliterator 1	6.965	Ras GTPase-activating-like protein IQGAP3	11.358
Protein TANC1	6.833	Epididymal secretory protein E1	9.054
Acetyl-CoA acetyltransferase, cytosolic	6.364	Integrin alpha-2	8.955
Signal transducer and activator of transcription 3	5.631	Growth/differentiation factor 15	8.3022
Elongation factor 1-alpha 2	5.212	Protein-glutamine gamma-glutamyltransferase 2	8.283
Superoxide dismutase [Mn], mitochondrial	4.863	PDZ and LIM domain protein 7	7.297
Farnesyl pyrophosphate synthase	4.605	Melanotransferrin	6.855
**Upregulated in WM115 Cells**		**Upregulated in WM266-4 Cells**	
**Proteins**	**Fold Change**	**Proteins**	**Fold Change**
Macrophage-capping protein	526.300	Serine/threonine-protein kinase DCLK1	28.759
Glyceraldehyde-3-phosphate dehydrogenase, testis-specific	37.595	Melanotransferrin	10.494
Cytoglobin	7.445	LIM domain only protein 7	9.851
Basement membrane-specific heparan sulfate proteoglycan core protein	7.287	CD109 antigen	7.568
Fibronectin	5.340	Fatty acid-binding protein, brain	6.679
Condensin complex subunit 1	4.970	Protein S100-A13	5.543
Structural maintenance of chromosomes protein 2	4.576	3-ketoacyl-CoA thiolase, mitochondrial	5.407
Cathepsin L1	4.179	Ras-related protein Rab-8B	3.668
Myoferlin	3.939	Band 4.1-like protein 3	3.623
Squalene synthase	3.245	Integrin alpha-6	3.529
**Upregulated in WM115 Exosomes**		**Upregulated in WM266-4 Exosomes**	
**Proteins**	**Fold Change**	**Proteins**	**Fold Change**
Plexin-A1	7.044	Matrilin-2	35.037
Transferrin receptor protein 1	6.849	Collagen alpha-1(VI) chain	25.929
Annexin A1	6.160	Extracellular matrix protein 1	23.545
Tyrosine--tRNA ligase, cytoplasmic	6.085	C-type lectin domain family 11 member A	17.627
60S ribosomal protein L5	5.851	Adipocyte enhancer-binding protein 1	17.042
60S ribosomal protein L7a	5.456	Procollagen-lysine,2-oxoglutarate 5-dioxygenase 1	16.935
Erythrocyte band 7 integral membrane protein	5.422	Semaphorin-5A	14.349
60S ribosomal protein L7	5.390	Vitamin K-dependent protein S	14.241
Sodium/potassium-transporting ATPase subunit beta-1	5.368	Tenascin	13.403
A-kinase anchor protein 12	5.131	Agrin	11.801

## Data Availability

MS/MS data are available via ProteomeXchange with identifier PXD038861.

## Supplementary Materials

The following supporting information can be downloaded at: https://www.mdpi.com/article/10.3390/cancers15041097/s1, Supplementary Data S1: Original Blots; Supplementary Data S2: Proteins lists/Gene Ontology Results; Supplemenatary Data S3: LFQ analysis of identified proteins/Interactome Diagrams.

## Abbreviations

CM	Cutaneous Melanoma
EV	Extracellular Vesicles
LC–MS/MS	Liquid Chromatography and Mass Spectrometry
NTA	Nanoparticle Tracking Analysis
