# Supplementary figures and images for: Similarities and Differences in the Protein Composition of Cutaneous Melanoma Cells and Their Exosomes Identified by Mass Spectrometry
Source: Cancers (Basel). 2023 Feb 8;15(4):1097. doi: 10.3390/cancers15041097 (PMC9954195; doi:10.3390/cancers15041097)

Original blots from Figure 2

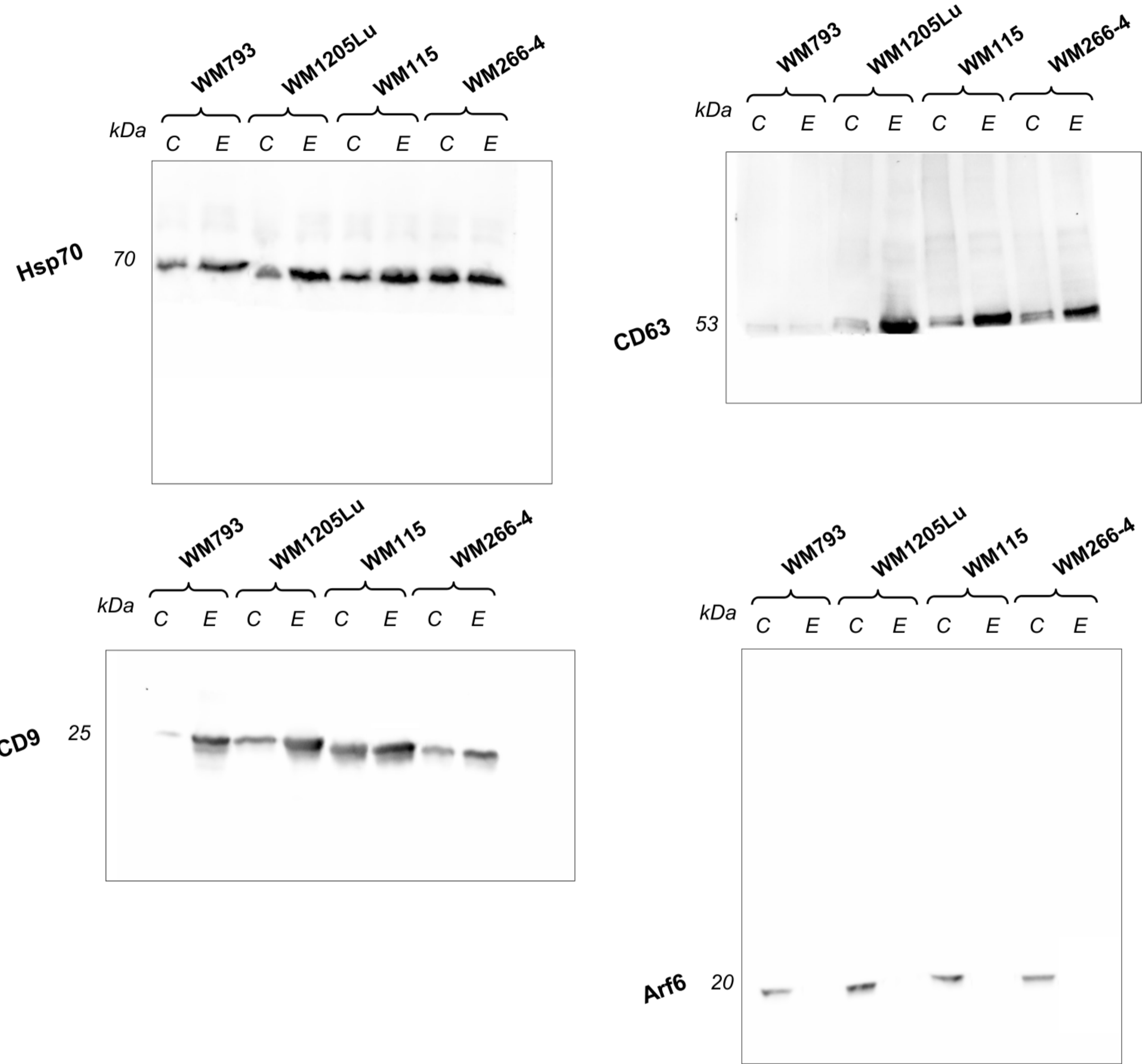

Supplement: Supplementary file 1 [file cancers-15-01097-s001.zip › Supplementary Data/Supplementary Data S1 Original Blots.pdf]
